# Uncovering Hidden Phenotypes in NEX‐Cre Mice: Behavioral and Cellular Alterations Demand Re‐Evaluation of a Widely Used Transgenic Line

**DOI:** 10.1111/jnc.70401

**Published:** 2026-03-15

**Authors:** Kim Renken, Olivia Andrea Masseck

**Affiliations:** ^1^ Synthetic Biology, University of Bremen Bremen Germany; ^2^ Neuromodulatory Circuits University of Cologne Cologne Germany

**Keywords:** Cre‐recombinase, NEX‐Cre, spine density, transgenic models

## Abstract

Transgenic mouse strains are essential tools in neuroscience, enabling targeted genetic manipulations to investigate brain function and neurological diseases. The NEX‐Cre mouse line, which targets glutamatergic principal neurons in the neocortex and hippocampus by expressing Cre‐recombinase under the NEX (NeuroD6) promoter, has been widely used for conditional gene manipulation. Contrary to previous reports suggesting no behavioral and histological abnormalities in NEX‐Cre mice, our study reveals distinct behavioral and cellular phenotypes. Behavioral analyses indicate reduced anxiety‐like behavior, altered reward‐related behavior, and increased locomotor activity in NEX (Cre/Cre) mice. Additionally, Support Vector Machine (SVM) analysis uncovered subtle strain‐specific and genotype‐specific behavioral traits across all NEX‐Cre genotypes relative to the commonly used C57BL/6J mouse strain. While overt behavioral abnormalities were most prominent in NEX (Cre/Cre) mice, SVM‐based analysis revealed subtle genotype‐ and strain‐specific behavioral signatures across NEX‐Cre genotypes. This underlines the importance of using littermate controls rather than independently maintained or purchased C57BL/6J animals when interpreting genotype‐related effects. Histological analyses of Golgi‐Cox‐stained brain slices revealed alterations in dendritic spine density across key brain regions, including the caudate putamen, hippocampal CA1, nucleus accumbens core region, lateral septum, and medial prefrontal cortex. These findings highlight significant inter‐ and intra‐strain variability, emphasizing the importance of careful characterization of transgenic models and the need for appropriate control groups and experimental designs to ensure the reliability and validity of studies utilizing Cre‐Driver lines.

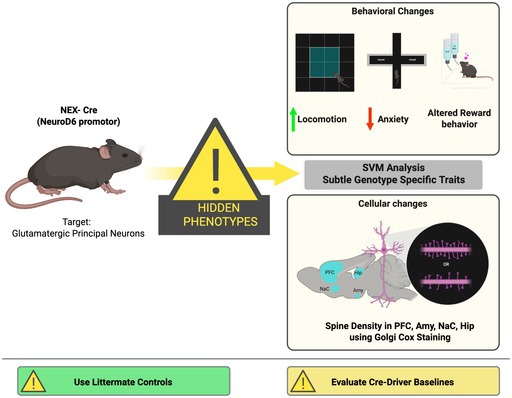

## Graphical abstract

Cre‐driver mouse lines are widely used for targeted gene manipulation, yet their baseline phenotypes are often assumed to be neutral. Here, we reveal hidden behavioral and synaptic alterations in the commonly used NEX‐Cre line, including changes in locomotion, anxiety‐like behavior, reward‐related behavior, and region‐specific dendritic spine density. Machine‐learning analysis further detects subtle genotype‐dependent behavioral signatures across NEX‐Cre genotypes compared with C57BL/6J controls. These findings highlight the need to evaluate Cre‐driver baselines and to prioritize littermate‐controlled experimental designs.

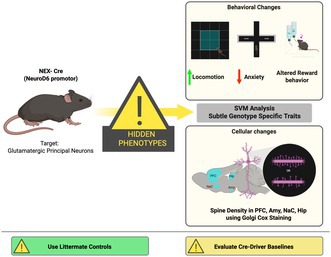

**Abbreviations**
bHLH: basic Helix‐loop‐Helic
BLA: basolateral amygdala
CA1: Cornu ammonis 1
CNS: central nervous system
CPu: caudate putamen
Cre: Cre Recombinase
EPM: elevated plus maze
LSD: dorsal lateral septum
LSO: intermediate lateral septum
mPFC: medial prefrontal cortex
NaC: nucleus accumbens core region
NEX: NeuroD6
NSFT: novelty suppressed feeding test
OFT: openfield test
PCA: principal component analysis
SHAP: SHapley Additive exPlanations
SI: social interaction
SPT: sucrose preference test
SVM: support vector machine
t‐SNE: t‐distributed stochastic neighbor embedding
VTA: ventral tegmental area

## 1 Introduction

Transgenic mouse models are indispensable tools for investigating the highly complex mechanisms underlying central nervous system (CNS) functions. They enable precise genetic manipulations to study specific molecular pathways and cellular processes in vivo, providing critical insights into neurodevelopment, behavior, and disease. Transcription factors are essential regulators of neurodevelopment, orchestrating processes like neuronal differentiation, mitochondrial function, and neuroprotection. The transgenic NEX‐Cre mouse line expresses Cre recombinase under the control of the NEX promoter, specifically targeting glutamatergic principal neurons in the neocortex and hippocampus (Goebbels et al. 2006). This Cre line is constitutive, enabling continuous Cre‐recombinase activity throughout development and adulthood. The NEX‐Cre mouse line was developed by Goebbels et al. (2006) and is widely used for conditional gene manipulation in pyramidal neurons of the dorsal telencephalon to study cortical development, learning, and memory.

The NEX gene was originally identified as NEX‐1 (Bartholomä and Nave 1994) but later became known under several other names, including MATH‐2, ATOH‐2, or NeuroD6. Although the NEX gene is now commonly referred to as NeuroD6, we retain the term “NEX‐Cre” to align with the original nomenclature under which this transgenic mouse line was first described (Goebbels et al. 2006) and widely recognized in the scientific literature.

As a member of the NeuroD family of basic Helix–loop‐Helic (bHLH) transcription factors, NEX plays a key role in neuronal differentiation, mitochondrial dynamics, and synaptic function. The NeuroD family is characterized by overlapping spatiotemporal expression patterns in the dorsal telencephalic neuroepithelium during early development, and a certain degree of functional redundancy is assumed among these factors (Schwab et al. 1998; Oproescu et al. 2021; Tutukova et al. 2021). The assumption of functional redundancy between the members of the NeuroD family, in combination with the specific expression of NEX in forebrain glutamatergic principal neurons and the non‐lethal effects of NEX deficiency, made NEX an ideal target gene for the generation of NEX‐Cre animals (Goebbels et al. 2006; Schwab et al. 1998). However, the knock‐in of Cre‐recombinase into the NEX locus by homologous recombination in embryonic stem cells renders the NEX gene permanently nonfunctional, raising concerns about the potential developmental consequences. NEX regulates anti‐apoptotic factors, molecular chaperones, and reactive oxygen species metabolism, which are essential for neuronal survival (Uittenbogaard and Chiaramello 2005; Uittenbogaard et al. 2010a, 2010b). NEX contributes to cytoskeletal remodeling, a process essential for dendritic spine formation, synaptic plasticity, and overall neuronal health (Gu et al. 2008). In addition to its expression in glutamatergic principal neurons of the neocortex and hippocampus (Goebbels et al. 2006; Schwab et al. 1998), it is also expressed in a subpopulation of midbrain dopaminergic (mDA) neurons in the ventral tegmental area (VTA), which project to the nucleus accumbens shell (Khan et al. 2017; Kramer et al. 2021).

Optogenetic activation of NEX‐expressing mDA neurons in the VTA induces dopamine release, glutamatergic postsynaptic responses, and real‐time place preference (Bimpisidis et al. 2019), while silencing impairs dopamine release and leads to behavioral abnormalities, such as increased consummatory behavior without changes in motivation for sucrose rewards (Bimpisidis et al. 2023). While these data suggest a role for NEX in behavioral modulation, the precise impact of genetic modifications in NEX‐Cre animals on their behavior remains unclear. Initial reports about the NEX‐Cre mice indicated no apparent histological or behavioral abnormalities (Goebbels et al. 2006; Schwab et al. 1998). While some studies reported reduced anxiety‐like behavior, mild anhedonic‐like behavior, motor abnormalities, and learning deficits, others did not observe such abnormalities (Berg 2019; Mikhailova 2007; Loganathan et al. 2024).

Transgenic mouse strains, including NEX‐Cre mice, often exhibit behavioral or cellular abnormalities that might complicate data interpretation. These issues may arise from gene overexpression, gene knockout or off‐target effects, which can unexpectedly affect protein levels, cellular functions, and downstream pathways and might often lead to pleiotropic effects and contribute to the development of neurodevelopmental and neurodegenerative diseases (Parenti et al. 2020). Insertional mutagenesis and compensatory mechanisms may further lead to phenotypic changes, and even the genetic background of the strain introduces variability into experimental outcomes. Cre‐recombinase itself has been associated with behavioral changes, such as hyperactivity and impulsivity, as well as developmental defects (Schmidt et al. 2000; Desor et al. 2024). Therefore, thorough characterization of transgenic mouse lines is critical to prevent misinterpretation or incorrect attribution of observed phenotypes to specific genetic modifications. This is particularly important when inherent behavioral abnormalities cannot be excluded and could potentially interfere with experimental results.

In the present study, the behavior and cellular architecture of male NEX‐Cre mice, bred on a C57BL/6J background, including heterozygous (+/Cre) and homozygous (Cre/Cre) mutants as well as wild‐type littermates (+/+), were characterized and compared to male mice of the commonly used C57BL/6J mouse strain. Our findings reveal clear behavioral abnormalities in NEX‐Cre (Cre/Cre) homozygous mice, including reduced anxiety‐like behavior, hyperlocomotion, motor deficits, and spine density abnormalities in key brain regions such as the nucleus accumbens, hippocampus, and caudate putamen. Spine density abnormalities were also observed in NEX‐Cre (Cre/Cre) and NEX‐Cre (+/Cre) mice.

## 2 Methods

### 2.1 Experimental Model and Subject Details

#### 2.1.1 Animals

Adult male naïve wildtype (+/+), heterozygous NEX (+/Cre), and homozygous NEX(Cre/Cre) NEX‐Cre mice were bred on a C57BL/6J background using a NEX(+/Cre) × NEX (+/Cre) pairing (Goebbels et al. 2006). The NEX‐Cre mouse line was originally generated by the Nave lab and colleagues and (Goebbels et al. 2006) was obtained as a gift from the Nave laboratory at the Max Planck Institute of Experimental Medicine (Göttingen, Germany). Although congenicity was not confirmed by SNP genotyping, the line fulfills the criterion of ≥ 10 backcross generations on a C57BL/6J background. The residual 129‐derived sequence surrounding the NEX locus has not been mapped explicitly. However, all experiments were performed using Cre‐negative wild‐type littermates as controls, which controls for any potential background effects linked to the insertion site. Male C57BL/6J mice (The Jackson Laboratory) were used as inter‐strain control. All mice used in this study were between 2 and 9 months of age. Animals were maintained on a standard 12‐h light/dark cycle and housed in groups within individually ventilated cages (IVC, Zoonlab) under controlled conditions (22°C ± 2°C, 50% ± 5% humidity). Food and water were provided *ad libitum*. Mice were housed in groups of 2–4 animals per cage until the beginning of behavioral testing. Two days prior to the start of the experiments, mice were single‐housed to allow habituation and to ensure consistency in behavioral testing conditions. All experiments were conducted during the dark phase, aligning with the animals' primary activity period. Anxiety tests were performed with a 1‐week interval. All procedures followed the guidelines set by the Senator für Gesundheit, Frauen und Verbraucherschutz of the Freie Hansestadt Bremen.

## 3 Method Details

### 3.1 Key Resources Table

**Table:** 

Reagent or resource	Source	Identifier
Critical commercial assays		
FD Rapid GolgiStain Kit	FD NeuroTechnologies, Ellicott City	Cat. No: t# PK401
Experimental models: Organisms/strains		
C57BL/6J	The Jackson Laboratory	RRID:IMSR_JAX:000664
NEX‐Cre (or NeuroD6‐Cre)	Laboratory of Prof. Nave ^1^	N/A
Hsd: ICR (CD‐1) outbred mice, ex‐breeder	Inotiv (Envigo)	RRID:IMSR_CRL:022—*Crl:CD1(ICR) mouse, outbred stock* *Cat. No*. INOTIV:030
Software and algorithms		
Python (version 3.10.1)	Python Software Foundation	RRID:SCR_008394 https://www.python.org/
EthoVision XT	Noldus (Virgina, USA)	RRID:SCR_000441 Version: 15.0.141
GraphPad Prism	GraphPad Software	RRID:SCR_002798 Version: 9.3.1.

#### 3.1.1 Behavioral Experiments

##### 3.1.1.1 Elevated Plus Maze (EPM)

The EPM is a widely used behavioral test to assess anxiety‐like behavior, locomotor, and exploratory activity in rodents. This test leverages the natural exploratory behavior of mice and their aversion to open and elevated areas. The EPM consists of two open arms (33.5 cm in length, 5 cm wide) and two closed arms (33.5 cm in length, 17 cm high wall, 5 cm wide) connected by a central platform, elevated 43 cm above the ground. Experiments were conducted under bright lighting conditions (~900 lx). Mice were individually transported from the animal facility to the experimental room and placed at the center of the maze, facing the open arm opposite to the experimenter. The behavior of each mouse was recorded for 5 min. Data were analyzed using EthoVision XT software (Noldus), with automated tracking quantifying the time spent in each zone, entrances to each zone, total distance, and velocity.

##### 3.1.1.2 Open Field Test (OFT)

The OFT is a commonly used behavioral assay to evaluate anxiety‐like behavior, locomotion, and exploratory activity in rodents. The OFT was conducted under bright lighting conditions (~900 lx) and takes place in a 50 cm (width) × 50 cm (depth) × 50 cm (height) Plexiglas arena. The arena was virtually divided into 16 equal squares, with the 4 inner squares representing the center zone (25 cm × 25 cm). Mice were individually transported from the mouse facility to the experimental room. Each mouse was placed in the lower left corner of the arena, facing the center. Behavior was recorded for 5 min and videos were analyzed using EthoVision XT software (Noldus). Automated tracking quantified the time spent in each zone, entrances to each zone, total distance, and velocity. Time spent in the center zone and center zone entries are inversely correlated with anxiety levels. Circling behavior was tracked automatically using EthoVision XT software by counting body axis rotation which was defined as 360° rotation around the axis of the center point and nose point. Offline analysis of grooming and rearing behavior in the open field test was performed by a trained observer using predefined scoring criteria. Rearing was defined as the mouse standing on its hind paws with the forelimbs lifted off the ground.

##### 3.1.1.3 Novelty Suppressed Feeding Test (NSFT)

For the NSFT, mice were transferred to a new cage and food‐deprived for 24 h prior to testing. The test was conducted in the open field arena, which was filled with approximately 200 g of fresh bedding per animal. A piece of filter paper (5 cm × 5 cm) with a standard food pellet was placed on the bedding in the center of the arena. Food pellets were weighed at the start of the experiment. The NSFT comprised three phases: habituation, test, and feeding. During the habituation phase, each animal was acclimated to the lighting conditions and the experimental room for 5 min. In the test phase, each mouse was placed in the back left corner of the open field arena, facing the food pellet. The test was terminated once the animal grasped the pellet with both front paws and began eating, with a maximum duration of 10 min allowed. The latency to begin eating was recorded. Following the test phase, each mouse was returned to its home cage and brought back to the mouse facility for the feeding phase, conducted immediately afterward. In this phase, each mouse had 5 min to eat the food pellet in the dark. The pellet was then weighed to calculate total food intake. After the experiment, all mice were given *ad libitum* access to food. The habituation and test phases were conducted under bright lighting conditions (~900 lx), while the feeding phase took place in darkness. Throughout the experiment, mice had *ad libitum* access to water. Latency to begin feeding is positively correlated with anxiety, while food intake serves as a control for normal feeding behavior.

##### 3.1.1.4 Sucrose Preference Test (SPT)

The SPT is a two‐bottle choice test used to assess anhedonic behavior, a core symptom of depression. This test is conducted in the home cage of each mouse during the active phase of the animals. For this experiment, 125 mL glass bottles were used, each equipped with a neck containing a small metal ball to prevent dripping during setup. The SPT consists of two phases: the habituation phase and the test phase. In the habituation phase, each mouse was given access to two bottles of tap water for 48 h. Following this, the water bottles were replaced with two bottles containing freshly prepared 1% sucrose solution (w/v) for another 48 h. During the test phase, each mouse had access to one bottle containing tap water and one bottle of 1% sucrose solution for 6 h. Bottles were weighed before and after the test to calculate sucrose preference as a percentage of total liquid intake.

##### 3.1.1.5 Social Interaction Test (SIT)

SIT is a behavioral assay used to assess social behavior and interactions in mice. This test was conducted in the open field arena (50 cm × 50 cm), where a small perforated Plexiglas chamber (10 cm wide × 6.5 cm deep × 42 cm high) was placed along one side of the arena. An area around this enclosure (12.5 cm × 25 cm) was defined as the social interaction area. The experiment consists of three phases: habituation, exploration, and interaction, following the protocol established by Golden and colleagues in 2011 (Golden et al. 2011). In the habituation phase, each mouse was individually brought to the experimental room and acclimated under red‐light conditions for 1 h. In the exploration phase, each mouse was placed at the center of the wall opposite the Plexiglas chamber in the open field arena and allowed to explore for 150 s before being returned to its home cage. For the interaction phase, an unfamiliar mouse (adult male CD‐1 mouse) was placed in the Plexiglas chamber. Each mouse was placed again at the center of the wall opposite the chamber and allowed to explore the arena and interact with the unfamiliar mouse in the chamber for 150 s (Golden et al. 2011). The social interaction ratio (SI‐ratio) is calculated by dividing the time spent in the interaction zone when the unfamiliar mouse is present by the time spent in the interaction zone when the unfamiliar mouse is absent. An SI‐ratio of 1 indicates that the mouse spent equal time in the interaction zone during presence and absence of a social target. In addition, the time that each mouse spends in the interaction zone during the interaction phase was calculated as a percentage and given as the socialization time.

The variable sample sizes across behavioral assays reflect the fact that experiments were conducted in several independent cohorts. Within each cohort, animals from all genotypes were tested together, using the same fixed inter‐test intervals, ensuring that prior testing experience did not systematically differ between genotypes. No pre‐determined inclusion or exclusion criteria were applied. For the NSFT, three animals (1 NEX(+/+), 1 NEX(Cre/+), and 1 NEX(Cre/Cre)) were excluded because their latency to feed exceeded 100 s. No other animals were excluded from any analyses. All remaining animals that entered a given experiment were included in the final datasets, and no animals died during the course of the experiments. No a priori sample size calculation was performed. Sample sizes were determined based on previous studies using similar behavioral and morphological assays and on practical considerations related to animal availability, with all animals from the respective cohorts included in the analyses.

#### 3.1.2 Histology

##### 3.1.2.1 Gelatine‐Coated Microscope Slides

Microscope slides were polished, placed in a staining rack, soaked overnight in detergent and ddH_2_O, rinsed, and air‐dried at room temperature. The following day, the slides were cleaned in an ultrasonic bath with filtered isopropanol for 15 min, followed by immersion in boiling filtered 96% ethanol for 2 min. After drying at room temperature overnight, the slides were ready for gelatin coating. For coating, powdered gelatin was dissolved in ddH_2_O to prepare a 0.5% solution (w/v) and heated to 70°C. Chromium potassium sulfate (CrK(SO_4_)_2_) was added to achieve a 0.05% solution (w/v), and the mixture was stirred until the color changed from yellow to green‐blue. The solution was filtered and maintained at 75°C. For the first coating, cleaned slides were slowly dipped into the gelatin solution, covered and dried at room temperature overnight. The next day, a second coating was applied using freshly prepared gelatin‐chromium potassium sulfate solution. The coated microscope slides were stored covered until use.

##### 3.1.2.2 Perfusion and Golgi‐Cox Staining

Following behavioral experiments, animals were euthanized via a lethal intraperitoneal injection of a ketamine/xylazine cocktail (130 mg/kg ketamine, 10 mg/kg xylazine). The mice were transcardially perfused with 1× phosphate‐buffered saline (PBS) for 15 min, and their brains were stored for 24 h at 4°C in a 30% sucrose solution (w/v) for cryoprotection.

Dendritic spine density was determined using Golgi‐Cox staining, a widely used method for visualizing neuronal morphology, including dendritic spines. In this study the FD Rapid GolgiStain Kit (*Cat. No*.: PK401, FD NeuroTechnologies, Ellicott City) was used, following the manufacturer's instructions. After impregnation, the brains were snap‐frozen at −70°C and stored at −80°C until the next day. On the following day, the brain tissue was mounted onto specimen discs by applying thin layers of distilled water using a paintbrush on dry ice. The brains were cut into 100 μm thick sections using a cryostat. Brain sections were mounted directly onto gelatin‐coated glass slides and dried overnight at room temperature. The next day, the sections were stained according to the manufacturer's instructions, coverslipped with Eukitt, and stored at room temperature in the dark.

#### 3.1.3 Spine Density Analysis

Spine density was analyzed in male mice from four groups: C57BL/6J, NEX‐Cre (+/+), NEX‐Cre (wt/−), and NEX‐Cre (Cre/Cre). Golgi‐Cox staining was used to visualize dendritic spines in selected brain regions: mPFC (apical and basal), NaC, CPU, LSD, LSI, hippocampal CA1 (apical and basal), and BLA.

From each animal, multiple dendrites were selected using the following criteria: (1) clear Golgi impregnation without overlap from other structures, (2) dendritic segment ≥ 25 μm in length, and (3) location within the defined anatomical region. To avoid pseudoreplication, each dendrite was selected from a different neuron, and no two dendrites from the same cell were included. Quantification was performed blinded to strain and genotype, with this information added to the dataset only after the analysis was completed to ensure unbiased assessment.

Brightfield images were acquired with a 100× oil immersion objective (Leica Microsystems), and image stacks were processed using FIJI/ImageJ (Dendritic Spine Counter plugin). Spine density was calculated as the number of spines per 10 μm dendritic length. The total numbers of analyzed animals, brain slices, and dendrites per region and genotype are provided in Table S1.

## 4 Quantification and Statistical Analysis

All data analyses were performed in Python using the scikit‐learn package (Pedregosa et al. 2012).

### 4.1 Behavioral Cluster Identification

#### 4.1.1 PCA

**FIGURE 1:**
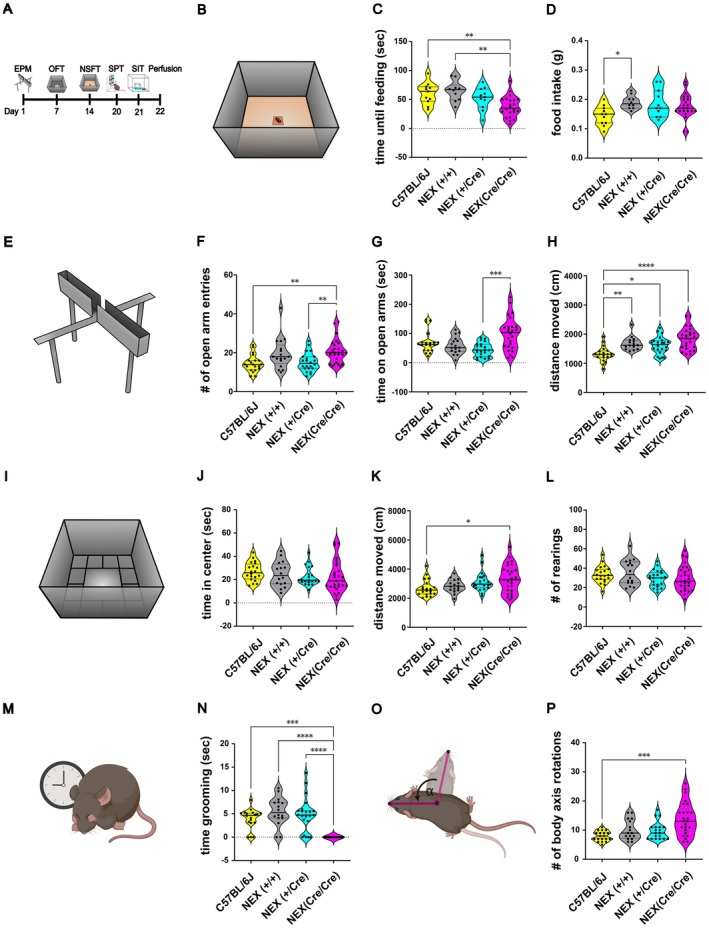
Altered anxiety‐like behavior and locomotor activity in NEX‐Cre mice compared to C57BL/6J controls in the EPM, OFT, and NSFT. (A) Overview of the behavioral test battery performed (please note not all animals underwent all tests) (B) Schematic overview of the NSFT. (C) In NSFT, NEX (Cre/Cre) showed a significantly decreased time until feeding compared to C57BL/6J mice and NEX (+/+) (Ordinary one‐way ANOVA: *F* (3, 50) = 6.93, ***p = 0.0005; Tukey's post hoc: C57BL/6J versus NEX (Cre/Cre) **p = 0.0065, NEX(+/+) versus NEX (Cre/Cre) **p = 0.0014). Sample sizes: C57BL/6J: *n* = 11, NEX(+/+): *n* = 10, NEX(+/Cre): *n* = 11, NEX (Cre/Cre): *n* = 22. (D) During the feeding phase of the NSFT, NEX (+/+) ate significantly more food compared to the C57BL/6J mice (Ordinary one‐way ANOVA: *F* (3, 50) = 3.128, *p* = 0.0338; Tukey's post hoc: C57BL/6J versus NEX (+/+) *p = 0.044). Sample sizes: C57BL/6J: *n* = 11, NEX (+/+): *n* = 10, NEX (+/Cre): *n* = 11, NEX (Cre/Cre): *n* = 22. (E) Schematic overview of the EPM. (F) Significantly increased open arm entries of the NEX (Cre/Cre) compared to the NEX (+/Cre) and the C57BL/6J mice in the EPM (Kruskal Wallis ANOVA: H (3) = 17.02, *p* = 0.0007; Dunn's post hoc: C57BL/6J versus NEX (Cre/Cre) **p = 0.0027, NEX‐Cre (+/Cre) versus NEX (Cre/Cre) **p = 0.0086). Sample sizes: C57BL/6J: *n* = 20, NEX (+/+): *n* = 17, NEX (+/Cre): *n* = 22, NEX‐Cre (Cre/Cre): *n* = 26. (G) NEX (Cre/Cre) spend significantly more time on the open arms of the EPM compared to the NEX (+/Cre) mice (Kruskal Wallis ANOVA: H (3) = 16.17, *p* = 0.001; Dunn's post hoc: NEX (+/Cre) versus NEX (Cre/Cre) ***p = 0.0005). Sample sizes: C57BL/6J: *n* = 20, NEX (+/+): *n* = 17, NEX(+/Cre): *n* = 22, NEX (Cre/Cre): *n* = 26. (H) Increased locomotor activity of NEX mice in the EPM compared to C57BL/6J mice (Kruskal–Wallis ANOVA: H (3) = 26.5, *p* < 0.0001; Dunn's post hoc: C57BL/6J vs. NEX (+/+) **p = 0.0046, C57BL/6J versus NEX(+/Cre) *p = 0.0195, C57BL/6J versus NEX (Cre/Cre) ****p < 0.0001). (I) Schematic overview of the OFT. Sample sizes: C57BL/6J: *n* = 20, NEX (+/+): *n* = 15, NEX(+/Cre): *n* = 22, NEX (Cre/Cre): *n* = 26. (J) All mice spend similar time in the center field in the OFT (Kruskal Wallis ANOVA: H (3) = 4.667, *p* = 0.1979). Sample sizes: C57BL/6J: *n* = 20, NEX (+/+): *n* = 16, NEX (+/Cre): *n* = 22, NEX‐ (Cre/Cre): *n* = 28. (K) Increased locomotor activity of NEX (Cre/Cre) mice in the OFT compared to C57BL/6J mice (Kruskal–Wallis ANOVA: H (3) = 9.572, *p* = 0.0226; Dunn's post hoc: C57BL/6J versus NEX (Cre/Cre) *p = 0.0247). Sample sizes: C57BL/6J: *n* = 20, NEX (+/+): *n* = 16, NEX(+/Cre): *n* = 22, NEX (Cre/Cre): *n* = 28. (L) All mice showed similar rearing behavior in the OFT (Ordinary one‐way ANOVA: *F* (3, 82) = 1.495, *p* = 0.2221). Sample sizes: C57BL/6J: *n* = 20, NEX (+/+): *n* = 16, NEX (+/Cre): *n* = 22, NEX (Cre/Cre): *n* = 28. (M) Schematic representation of grooming behavior. (N) NEX (Cre/Cre) spend significantly less time grooming compared to NEX‐Cre littermates and C57BL/6J mice during the OFT (Kruskal–Wallis ANOVA: H (3) = 35.22, *p* < 0.0001; Dunn's post hoc: C57BL/6J versus NEX (Cre/Cre) ***p = 0.0003, NEX (+/+) versus NEX‐ (Cre/Cre) ****p < 0.0001, NEX (+/Cre) versus NEX (Cre/Cre) ****p < 0.0001). Sample sizes: C57BL/6J: *n* = 18, NEX (+/+): *n* = 16, NEX (+/Cre): *n* = 22, NEX (Cre/Cre): *n* = 21. (O) Schematic representation of circling behavior tracking, where body axis rotations are determined based on the accumulation of rotation angles between the axes from the center point to the nose point. This method monitors micro‐rotations of animals turning around their own axis. A rotation is recorded when the cumulative angle of rotation (α) exceeds 360°. (P) During OFT, NEX (Cre/Cre) showed significantly increased circling behavior compared to C57BL/6J mice (Kruskal–Wallis ANOVA: H (3) = 17.99, *p* = 0.0004; Dunn's post hoc: C57BL/6J versus NEX (Cre/Cre) ***p = 0.0002). Sample sizes: C57BL/6J: *n* = 19, NEX (+/+): *n* = 16, NEX (+/Cre): *n* = 19, NEX (Cre/Cre): *n* = 25. The data is presented as individual data points on violin plot. The solid black line indicates the median, while the gray lines represent the 25th and 75th percentiles.

PCA (Principal Components Analysis) is a linear statistical technique used for dimensionality reduction by transforming a dataset into a set of orthogonal principal components while preserving essential information and reducing noise and computational complexity. This transformation helps reveal underlying patterns, clusters, or relationships within the data, making PCA particularly valuable for preprocessing in machine learning tasks (e.g., SVM) to mitigate overfitting and enhance model performance. In this study, two principal components were derived from a dataset comprising behavioral parameters from the EPM and OFT (Figure 1, Figures S2 and S3). The first principal component captures the direction of maximum variance in the data, representing the primary source of variability and illustrating the main axis of data distribution. The second principal component, orthogonal to the first, captures the next highest variance, reflecting the secondary axis of data spread. Together, these two components provide a simplified two‐dimensional representation of the dataset while retaining most of its original variability, facilitating effective visualization. PCA was conducted using Python.

#### 4.1.2 T‐SNE Analysis

t‐SNE (t‐Distributed Stochastic Neighbor Embedding) is a nonlinear dimensionality reduction technique designed for visualizing complex, high‐dimensional datasets. In this study, the input dataset consisted of PCA‐derived behavioral parameters from the EPM and OFT (Figure 1, Figures S2 and S3). t‐SNE operates by converting Euclidean distances between data points into conditional probabilities that indicate the likelihood of similarity between pairs of points. The algorithm then minimizes the divergence between the probability distributions of the high‐dimensional data and its low‐dimensional representation, ensuring that the resulting visualization accurately reflects local relationships within the data. This approach enables high‐dimensional data to be represented in a low‐dimensional space, making it easier to identify potential clusters or groups while preserving local structure (Van Der Maaten and Hinton 2008). t‐SNE was implemented using Python.

#### 4.1.3 SVM

SVM (Support Vector Machine) is a supervised machine learning algorithm widely used for classification tasks. In this study, PCA was applied as a preprocessing step for dimensionality reduction, providing a compact feature set and serving as the basis for an optimized SVM with a Radial Basis Function (RBF) kernel. This approach enhances the model's capacity to capture and process non‐linear relationships within the data efficiently. SVM constructs optimal hyperplanes in the transformed feature space to distinguish between different behavioral classes. To ensure the generalizability and robustness of the model, 5‐fold cross‐validation was employed, partitioning the dataset into multiple folds for training and testing on various subsets. Additionally, a bootstrap method was used to generate multiple resampled datasets, enabling the estimation of the model's variability and stability.

The combined use of PCA, SVM with RBF kernel, 5‐fold cross‐validation, and bootstrap resampling provides a rigorous framework for evaluating model performance, ensuring reliable and consistent behavioral classification outcomes. The aggregated confusion matrix summarizes classification results across all resampled datasets, offering insights into overall accuracy and the model's effectiveness in distinguishing behavioral classes. The SVM analysis was performed using Python.

In the analysis using the SVM algorithm, a comprehensive set of behavioral parameters from the OFT and EPM were used as input features. From the OFT, locomotion metrics included total distance moved (cm) and mean velocity (cm/s), along with spatial preferences and anxiety‐related behaviors such as frequency and total time spent in the border, corner, and center zones, as well as latency to first enter these zones. Additional metrics included frequency and total time of rearing, grooming, jumping, and body axis rotations. From the EPM, parameters included total distance moved (cm), mean velocity (cm/s), and exploratory measures such as closed and open arm entries, total time spent in closed and open arms, and latency to first enter these arms. The analysis also incorporated frequency and total time spent in the center field, along with latency to first enter the center. Together, these parameters provided a detailed behavioral profile, enabling the SVM algorithm to classify and interpret the data effectively (Figure 1, Figures S2 and S3).

#### 4.1.4 SHAP Analysis

SHAP (SHapley Additive exPlanations) analysis was applied to interpret the feature contributions to the predictions made by the SVM model. This method offers detailed insights into feature importance and interactions, enhancing the interpretability of complex models, such as SVMs with RBF kernels. Using the SHAP library in Python, SHAP values were computed for each feature across all samples, enabling both a global assessment of feature impact and individualized explanations of specific predictions. This dual‐level analysis provides a comprehensive understanding of how features influence the model's outputs, making the decision‐making process more transparent and interpretable.

### 4.2 Data Analysis

All behavioral data were recorded using EthoVision XT software from Noldus. Data acquisition was performed automatically from recorded videos. Statistical analysis and graphical illustrations were created using GraphPad Prism 9 software. Microscope images of Golgi‐Cox staining were processed for brightness and contrast using ImageJ. Quantification of dendritic spines was performed using ImageJ's dendritic spine counter plugin.

### 4.3 Statistical Analysis

Normality was assessed with the Shapiro–Wilk test, and homogeneity of variances across groups was evaluated using Bartlett's test. When both assumptions were met, a one‐way ANOVA was performed to determine whether the means of independent groups differed, followed by Tukey's post hoc test for pairwise comparisons. For non‐parametric data, the Kruskal‐Wallis ANOVA was used, followed by Dunn's post hoc test for group comparisons. Outliers were identified using the ROUT method of regression, which combines a robust nonlinear regression with a False Discovery Rate‐based test, employing a *Q*‐value of 1% to estimate the likelihood of false positive results (Motulsky and Brown 2006). No data points were excluded based on this analysis. Results are presented as violin plots with individual data points. Gray lines representing the 25th and 75th percentiles, solid black lines indicating the median. Significance levels were set at *p < 0.05, **p < 0.01, ***p < 0.001, and ****p < 0.0001.

## 5 Results

### 5.1 NEX (Cre/Cre) Mice Exhibit Reduced Anxiety‐Related Behavior and Increased Locomotor Activity

A comprehensive series of experiments was performed to characterize and compare the behavioral profiles of C57BL/6J, NEX (+/+), NEX (+/Cre), and NEX (Cre/Cre) mice (Figure 1A). To assess anxiety‐related behavior and general locomotion, three distinct behavioral paradigms were employed: the Novelty‐Suppressed Feeding Test (NSFT) (Figure 1B–D), the Elevated Plus Maze (EPM) (Figure 1E–H), and the Open Field Test (OFT) (Figure 1I–L). Among these, the NSFT primarily serves as a measure of anxiety‐like behavior, but it also provides insights into hunger, motivation to eat, and the animal's ability to overcome the conflict between the drive to feed and the aversiveness of a novel environment.

In the NSFT, NEX(Cre/Cre) mice initiated food consumption significantly faster than both NEX(+/+) and C57BL/6J mice (Figure 1C), suggesting a reduced anxiety‐like phenotype. Interestingly, NEX (+/+) mice consumed significantly more food than C57BL/6J controls (Figure 1D), which may indicate an alteration in consummatory behavior or metabolic regulation in the NEX‐Cre line.

Additional locomotor parameters, including total distance moved and mean velocity, are shown in Figure S1. NEX (Cre/Cre) mice traveled significantly shorter distances compared to both NEX (+/+) and NEX (+/Cre) mice. In contrast, NEX (+/+) and NEX (+/Cre) animals exhibited greater locomotor activity than C57BL/6J mice. Furthermore, NEX (Cre/Cre) mice displayed significantly lower mean velocity compared to both C57BL/6J and NEX (+/Cre) mice.

Similarly, in the EPM, NEX(Cre/Cre) mice entered the open arms significantly more frequently than both NEX (+/Cre) and C57BL/6J mice (Figure 1F) and spent significantly more time in the open arms compared to NEX (+/Cre) mice (Figure 1G). These findings are consistent with the NSFT results, further supporting a phenotype characterized by reduced anxiety‐like behavior and enhanced exploratory drive. Additionally, all NEX‐Cre genotypes exhibited significantly greater locomotor activity than C57BL/6J controls, as indicated by the increased total distance traveled during the EPM (Figure 1H). This observation suggests a general trend toward hyperactivity or elevated baseline locomotion in NEX‐Cre mice, irrespective of genotype.

NEX(Cre/Cre) mice exhibited particularly striking behaviors in the EPM, characterized by maladaptive movement patterns. During exploration of the open arms, these mice frequently slipped with their hind paws—even while running in a straight line—and displayed repetitive and erratic behaviors, such as circling on the open arms or the central platform. Their uncoordinated turning and frequent slipping occasionally led to partial or complete falls from the maze. Notably, these behaviors persisted throughout the testing session, with no evidence of habituation or corrective adaptation over time.

Quantitative analysis revealed that NEX (Cre/Cre) mice were significantly more likely to slip their hind paws off the open arms compared to NEX‐ (+/+), NEX (+/Cre), and C57BL/6J mice (Figure S2). While hind paw slips were occasionally observed in C57BL/6J, NEX (+/+), and NEX (+/Cre) mice, these events typically occurred only within the first few seconds after placement on the maze and were followed by rapid acclimation and confident locomotion. Among NEX (+/Cre) mice, behavioral variability was observed—some individuals showed frequent slipping, while others navigated the maze with ease.

Additional parameters, including mean velocity, locomotor trajectories, and detailed time spent in the open arms, closed arms, and central platform, are presented in Figure S2. Collectively, these findings further emphasize the phenotype of reduced anxiety‐like behavior, hyperactivity, and impaired motor coordination in NEX (Cre/Cre) mice.

In the OFT, all groups spent a similar amount of time in the center field (Figure 1J), and exhibited comparable frequencies of rearing behavior (Figure 1L), indicating no differences in anxiety‐like or exploratory behavior between the groups. However, increased locomotor activity was observed in NEX (Cre/Cre) mice compared to C57BL/6J mice, with NEX (+/+) and NEX (+/Cre) mice showing a slight trend toward increased locomotor activity (Figure 1K). Notably, NEX (Cre/Cre) mice exhibited striking behavioral abnormalities during the OFT. They did not display any self‐grooming behavior during the 5‐min test period, in contrast to the other groups (Figure 1N). This suggests a potential correlation between either the gene dosage of Cre recombinase or the NEX knockout and self‐grooming behavior. Additionally, similar to the EPM, NEX (Cre/Cre) mice demonstrated prominent circling behavior, rotating around their own axis significantly more often than C57BL/6J mice (Figure 1P). Additional details on behavioral parameters in the OFT are presented in Figure S3, including measurements of velocity, zone entries (border, corner, and center), corresponding latencies, as well as ethologically relevant behaviors such as rearing, circling, and grooming. NEX‐(Cre/Cre) mice entered the border zone significantly more frequently than both NEX (+/+) and C57BL/6J mice and spent more time in the border zones compared to C57BL/6J animals (Figure S3C,G). Moreover, both NEX (Cre/Cre) and NEX (+/Cre) mice spent less time rearing than C57BL/6J controls (Figure S3L), suggesting a reduction in vertical exploration.

Collectively, these findings support altered motor activity in NEX(Cre/Cre) mice, including changes in sequential movement patterns and the presence of stereotyped behaviors, distinguishing their behavioral phenotype from wild‐type NEX(+/+) and heterozygous counterparts. Overall, NEX‐Cre animals showed reduced anxiety‐related behavior and increased locomotor activity compared to C57BL/6J controls, suggesting that genetic background contributes to the gradual emergence of these behavioral differences. Notably, while NSFT and EPM indicated altered anxiety‐like behavior, the OFT did not provide clear evidence for such changes in NEX (Cre/Cre) mice, potentially reflecting differences in sensitivity and contextual demands across paradigms. Together, these results highlight the complex relationship between genetic alterations, locomotor activity, and anxiety‐related behavior, and emphasize the value of using complementary assays to capture multifaceted behavioral phenotypes.

### 5.2 NEX‐Cre Mice Show Normal Social and Mild Anhedonic‐Like Behavior

**FIGURE 2:**
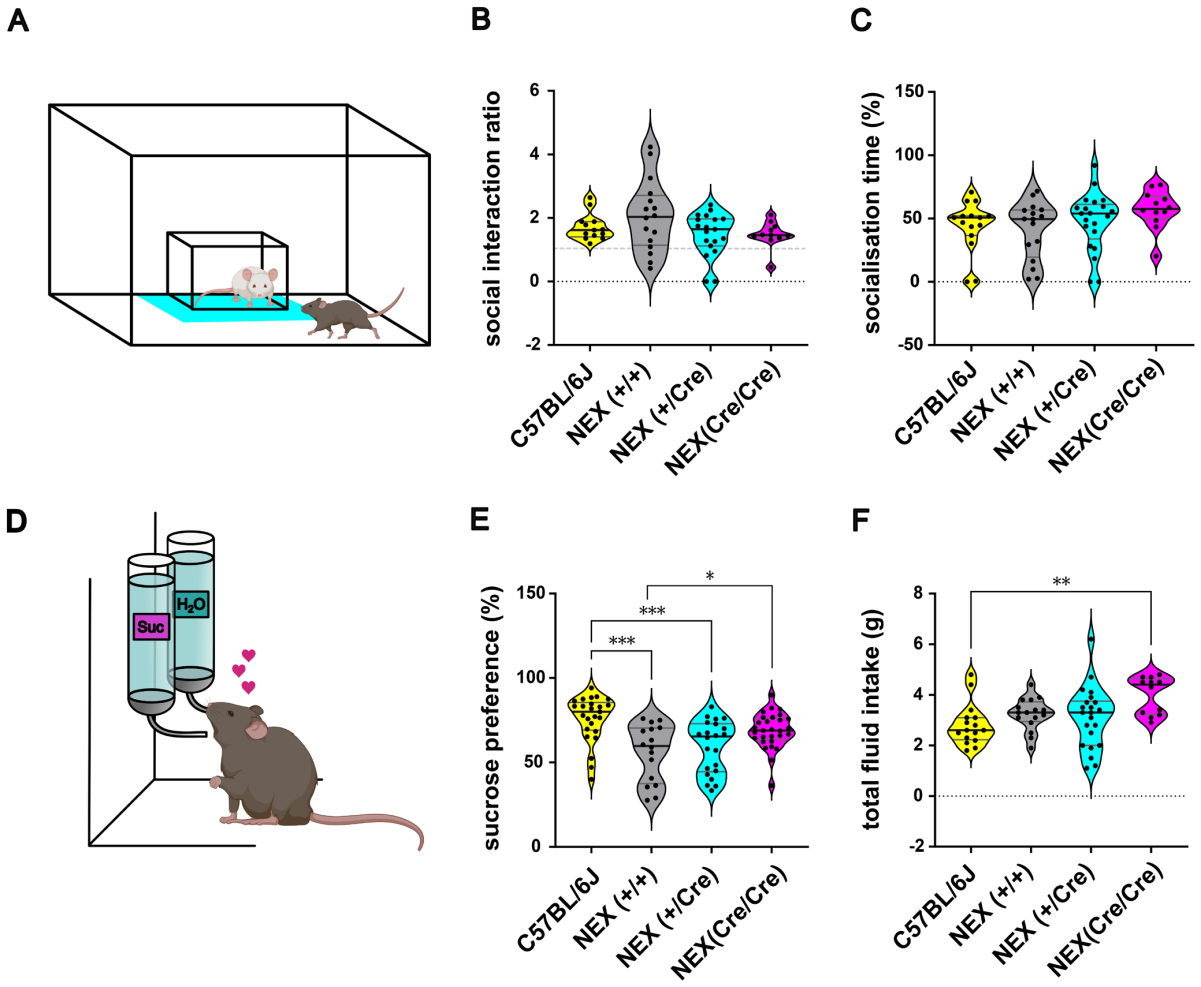
Decreased sucrose preference and normal social behavior in NEX‐Cre mice. (A) Schematic overview of the social interaction test. (B) SI‐ratios do not differ between the groups (Ordinary one‐way ANOVA: *F* (3, 55) = 2.061, *p* = 0.116). Sample sizes: C57BL/6J: *n* = 13, NEX (+/+): *n* = 16, NEX‐Cre (+/Cre): *n* = 19, NEX (Cre/Cre): *n* = 11. (C) No difference between the groups in the socialization time during social interaction test (Kruskal–Wallis ANOVA: H (3) = 4.941, *p* = 0.176). Sample sizes: C57BL/6J: *n* = 15, NEX (+/+): *n* = 16, NEX (+/Cre): *n* = 21, NEX(Cre/Cre): *n* = 12. (D) Schematic overview of the sucrose preference test. (E) Decreased sucrose preference in NEX‐ (+/+) and NEX (+/Cre) compared to C57Bl76J mice (Ordinary one‐way ANOVA: *F* (3, 88) = 9.376 ****p < 0.0001; Tukey's post hoc: C57BL/6J versus NEX‐Cre (+/+) ****p < 0.0001, C57BL/6J versus NEX (+/Cre) ***p = 0.0005, NEX (+/+) versus NEX (Cre/Cre) *p = 0.0174). Sample sizes: C57BL/6J: *n* = 25, NEX (+/+): *n* = 16, NEX (+/Cre): *n* = 22, NEX‐Cre (Cre/Cre): *n* = 29. (F) NEX(Cre/Cre) showed increased liquid intake compared to C57Bl76J mice (Kruskal–Wallis ANOVA: H (3) = 11.99, *p* = 0.0074; Dunn's post hoc: C57BL/6J versus NEX (Cre/Cre) **p = 0.0037). Sample sizes: C57BL/6J: *n* = 16, NEX(+/+t): *n* = 16, NEX (8Cre/+): *n* = 22, NEX (Cre/Cre): *n* = 12. The data is presented as individual data points on violin plot. The solid black line indicates the median, while the gray lines represent the 25th and 75th percentiles.

To assess social behavior in NEX (+/+), NEX (+/Cre), NEX (Cre/Cre), and C57BL/6J mice, we performed the social interaction (SI) test and calculated SI ratios (Figure 2A–C). No significant differences in SI ratios were observed between groups (Figure 2B). Notably, all C57BL/6J mice exhibited SI ratios above 1, whereas some mice in the NEX‐Cre groups fell below this threshold. Socialization time, defined as the percentage of time spent in the interaction zone during the interaction phase (Willmore et al. 2022), also did not differ between groups (Figure 2C).

In the sucrose preference test (SPT), NEX (+/+) and NEX (+/Cre) mice exhibited a significantly reduced sucrose preference compared to C57BL/6J mice (median values: C57BL/6J: 80%, NEX (+/+): 59.75%, NEX (+/Cre): 65.33%; *p* = 0.0005, *p* < 0.0001; ***, ****), suggesting innate anhedonic‐like behavior or altered reward sensitivity (Figure 2E). Interestingly, NEX (Cre/Cre) mice demonstrated a significantly increased sucrose preference compared to NEX (+/+) mice (median values: NEX (+/+): 59.75%, NEX (Cre/Cre): 68.89%; *p* = 0.0174; *), indicating heightened consummatory behavior relative to their wildtype littermates. Furthermore, during the 6‐h SPT, NEX (Cre/Cre) mice consumed significantly more sucrose and water than C57BL/6J mice (Figure 2F). Although the median fluid intake of NEX (+/+) and NEX (+/Cre) mice was slightly elevated, these differences did not reach statistical significance.

The social interaction test revealed no significant group differences, although some NEX‐Cre mice showed reduced social interest. NEX (+/+) and NEX (+/Cre) mice displayed lower sucrose preference, suggesting innate anhedonia or altered reward sensitivity. In contrast, NEX (Cre/Cre) mice showed increased sucrose preference and fluid intake, indicating heightened consumption and possibly elevated metabolism.

### 5.3 Classification Analysis of EPM and OFT Behavioral Data Reveal Subtle but Distinct Behavioral Differences Between NEX‐Cre Genotypes and C57BL/6J Mice

**FIGURE 3:**
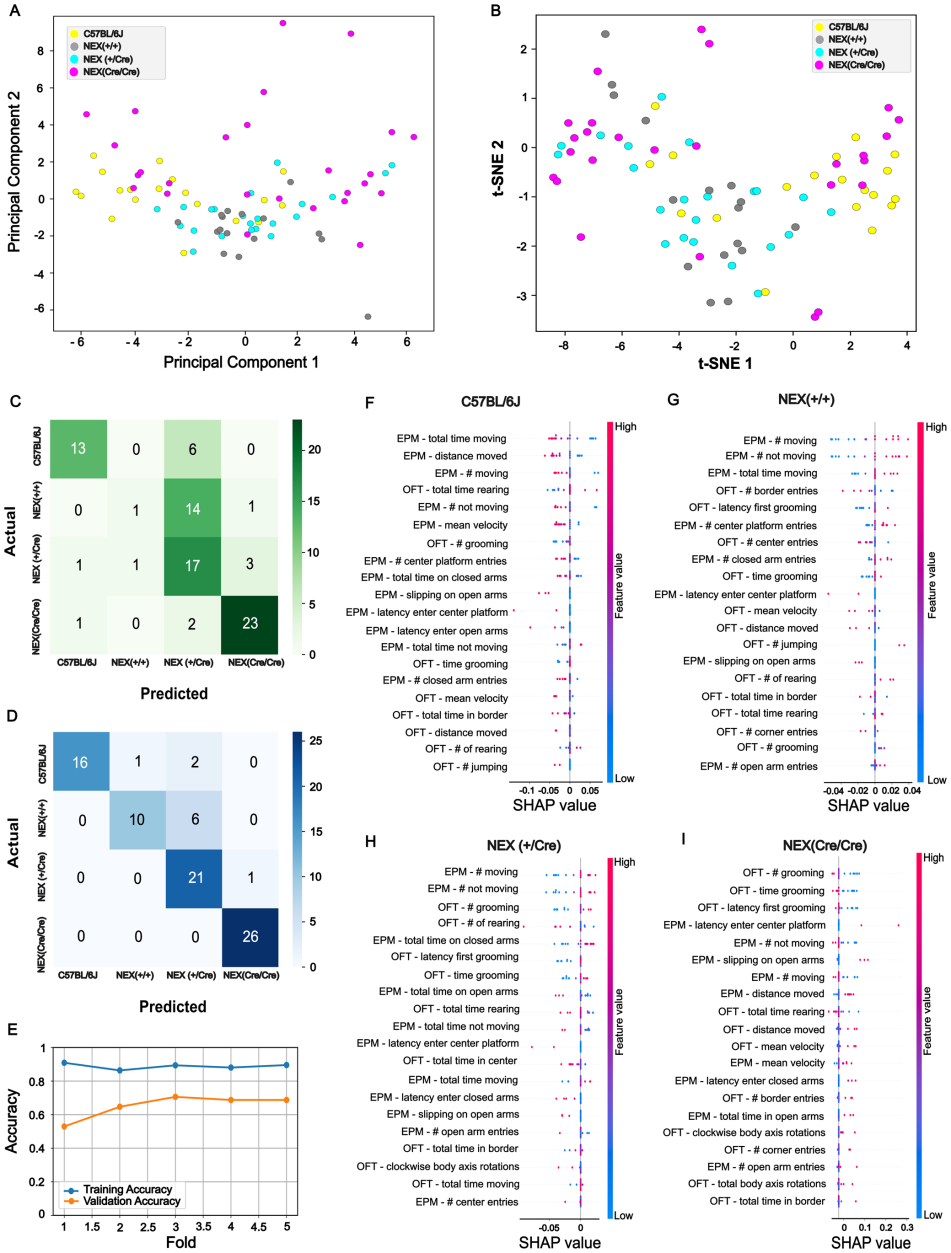
Dimensionality reduction and classification analysis of behavioral data from EPM and OFT: PCA, t‐SNE, SVM confusion matrix, and SHAP value insights. (A) Visualization of the dataset after transformation and dimensionality reduction using PCA, highlighting the primary patterns and capturing 38% of the variance in the dataset. (B) Visualization of the dataset preprocessed by PCA and then transformed and reduced to two dimensions using t‐SNE, with a silhouette score of −0.092 indicating strong cluster overlap, and a trustworthiness score of 0.85 reflecting a high level of preservation of local data structure. The silhouette score of −0.065 suggests strong overlap of the groups. (C) Aggregated confusion matrix using 5‐fold stratified cross‐validation reached a mean accuracy of ~65% and a F1‐score of ~0.6. (D) Confusion matrix of the entire dataset, mean accuracy 88%. (E) Model training and validation accuracy. (D–G) Beeswarm plot visualization of SHAP values derived from the SVM model, illustrating the contribution of features to the prediction of the groups: C57BL/6J (F), NEX (+/+) (G), NEX (+/Cre) (H), and NEX (Cre/Cre) mice (I). Each row represents a feature, ranked from top to bottom by the mean absolute SHAP value. Individual dots in each row represent SHAP values for that feature, with color indicating the feature value according to the color bar. The position along the x‐axis reflects the SHAP value, representing the feature's impact on the model's prediction. Sample sizes: C57BL/6J: *n* = 19, NEX (+/+): *n* = 16, NEX (+/Cre): *n* = 22, NEX (Cre/Cre): *n* = 26.

Although significant behavioral deficits were primarily observed in NEX (Cre/Cre) mice, we hypothesized that additional subtle differences might exist across genotypes that were not fully captured by traditional behavioral measures. To investigate this, we applied advanced machine learning techniques, including principal component analysis (PCA), t‐distributed stochastic neighbor embedding (t‐SNE), and support vector machine (SVM) classification, to uncover potential hidden behavioral patterns. Data from the OFT and EPM (Figure 1, Figures S2 and S3) served as the basis for these analyses, providing a comprehensive behavioral dataset for genotype classification. To assess whether spontaneous behavior alone is sufficient to differentiate between genotypes, we first applied PCA and t‐SNE to the behavioral feature set. Neither method revealed any clear clustering or separation by genotype (Figure 3A,B), suggesting that the behavioral differences are subtle and not linearly separable in the high‐dimensional feature space. PCA was first applied to obtain an overview of the data structure, capturing 38% of the total variance within the first two principal components (Figure 3A). The remaining unexplained variance (62%) likely reflects additional behavioral dimensions and interindividual variability that require higher‐order components or nonlinear methods to resolve. To further explore potential nonlinear relationships or subtle substructure in the behavioral data, we next applied t‐SNE (Figure 3B). The t‐SNE embedding achieved a trustworthiness score of 0.85, reflecting good preservation of local neighborhood structure in the high‐dimensional behavioral space. In contrast, the negative silhouette score (−0.065) indicated substantial overlap between genotypes, with no evidence for discrete behavioral clusters. In line with the PCA, these findings suggest that genotype‐dependent behavioral differences are subtle, multidimensional, and not linearly separable in the observed feature space.

To assess whether the behavioral alterations observed in NEX‐Cre mice extend beyond isolated group differences and form a coherent multivariate phenotype at the level of individual animals, we applied supervised classification to the combined OFT and EPM behavioral dataset. Despite the absence of clear structure in the unsupervised analyses, a supervised support vector machine (SVM) classifier was able to predict genotype based on the multivariate behavioral feature space with above‐chance accuracy. Using 5‐fold stratified cross‐validation, the model achieved a mean accuracy of approximately 65% and a mean F1‐score of 0.6. The aggregated confusion matrix exhibited clear diagonal dominance (Figure 3C), reflecting consistent genotype classification across folds. This performance was significantly above chance (31.3%; Binomial test, *p* < 0.001), indicating that genotype‐related behavioral signatures, although subtle, are systematically represented in the data. Evaluation on the full dataset yielded an overall accuracy of 0.88 (Figure 3D). The close agreement between training and validation accuracies (Figure 3E) confirms that the classifier generalizes well without signs of overfitting.

We next asked which behavioral features drive classification for each genotype. Using SHAP (SHapley Additive exPlanations), we visualized class‐wise feature importances based on the final fold of the SVM model. Each genotype exhibited distinct SHAP profiles, with different top‐ranked features contributing most to prediction (Figure 3F–I). SHAP feature attribution revealed both shared and class‐specific behavioral drivers of genotype classification (Figure 3F–I). For instance, the frequency of not moving in the EPM was consistently ranked among the top features across multiple genotypes but showed the strongest and most directional contribution to predictions of the NEX(+/Cre) group. In contrast, latency to first enter the center platform during EPM was an important class‐specific feature for identifying the NEX (Cre/Cre) group but contributed negligibly to predictions in the NEX (+/+) group. These results suggest that the classifier draws on both shared and genotype‐distinct behavioral signatures. Importantly, grooming behavior surfaced as a distinctive marker: grooming frequency was strongly weighted in the classification of NEX (Cre/Cre), consistent with the striking absence of this behavior in this group. Similarly, circling frequency, a marker often associated with stereotypy or motor abnormalities, as well as slipping in the EPM contributed more to predictions in NEX (Cre/Cre) than in other genotypes—further supporting the model's sensitivity to subtle motor behavioral changes. As expected based on the univariate analyses, grooming frequency, circling behavior, and slipping in the EPM contributed most strongly to classification of NEX (+/+) animals. Importantly, these features therefore not only differed at the group level but also carried high discriminative value for genotype prediction at the level of individual animals. In addition, the SHAP profiles revealed further genotype‐specific contributions from more subtle behavioral parameters, indicating that classification performance reflects an integration of both prominent and distributed behavioral signals.

In summary, the SVM classifier was able to reliably predict genotype from behavior with an accuracy well above chance, despite the absence of clear separability in unsupervised dimensionality reduction. SHAP analysis further uncovered distinct behavioral features that contributed to genotype classification, offering interpretable insights into subtle yet meaningful behavioral differences. These findings highlight the complexity of genotype‐specific behavioral signatures and underscore the role of both strain and genetic background in shaping behavior.

### 5.4 Structural Alterations of Dendritic Spine Density

**FIGURE 4:**
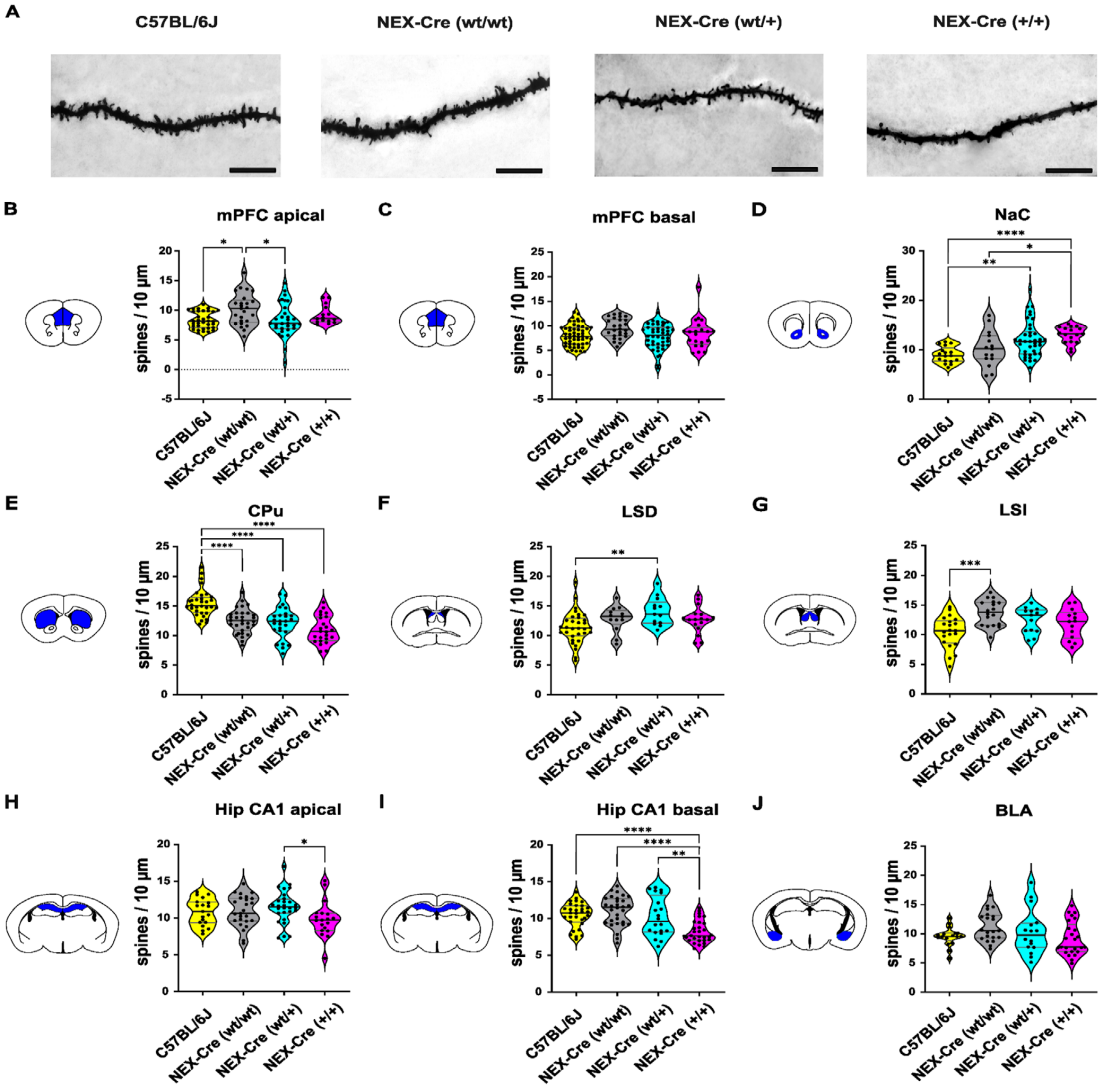
Dendritic Spine Density of NEX‐Cre and C57BL/6J mice. (A) Representative images of Golgi‐Cox‐stained basal dendrites of pyramidal neurons in the CA1 region of the hippocampus. Scalebar 10 μm. (B) Increased dendritic spine density on apical dendrites of pyramidal neurons of layer V in the PFC of NEX (+/+) mice compared to C57BL/6J and NEX (+/Cre) mice (Kruskal Wallis ANOVA: H (3) = 10.41, *p* = 0.0154; Dunn's post hoc: C57BL/6J versus NEX (+/+) *p = 0.0344, NEX‐(+/+) versus NEX (+/Cre) *p = 0.0498). Sample sizes of analyzed dendrites: C57BL/6J: *n* = 34, NEX (+/+): *n* = 25, NEX (+/Cre): *n* = 31, NEX (Cre/Cre): *n* = 15. (C) Spine density on basal dendrites in PFC (Kruskal–Wallis ANOVA: H (3) = 8.219, *p* = 0.0417, Dunn's post hoc: *p* values > 0.05). Sample sizes of analyzed dendrites: C57BL/6J: *n* = 52, NEX (+/+): *n* = 26, NEX (+/Cre): *n* = 44, NEX (Cre/Cre): *n* = 21. (D) Increased spine density on medium spiny neurons of the NaC of NEX (+/Cre) and NEX (Cre/Cre) mice compared and C57BL/6J mice but also in NEX (Cre/Cre) compared to NEX (+/+) mice. (Kruskal–Wallis ANOVA: H (3) = 22.61, *p* < 0.0001, Dunn's post hoc: C57BL/6J versus NEX (+/Cre) **p = 0.0029, C57BL/6J versus NEX (Cre/Cre) ****p < 0.0001, NEX (+/+) versus NEX(Cre/Cre) *p = 0.0258). Sample sizes of analyzed dendrites: C57BL/6J: *n* = 17, NEX (+/+): *n* = 15, NEX (+/Cre): *n* = 38, NEX (Cre/Cre): *n* = 17. (E) Decreased spine density on medium spiny neurons of the CPu in all Nex‐Cre groups compared to C57BL/6J mice (Ordinary one‐way ANOVA: *F* (3, 109) = 20.46, ****p < 0.0001; Tukey's post hoc: C57BL/6J versus NEX (+/+) ****p < 0.0001, C57BL/6J versus NEX (+/Cre) ****p < 0.0001, C57BL/6J versus NEX (Cre/Cre) ****p < 0.0001). Sample sizes of analyzed dendrites: C57BL/6J: *n* = 32, NEX‐Cre (+/+): *n* = 31, NEX (+/Cre): *n* = 26, NEX (Cre/Cre): *n* = 24. (F) Increased spine density on neurons the LSD in NEX(+/Cre) mice compared to C57BL/6J mice (Ordinary one‐way ANOVA: *F* (3, 71) = 4.056, *p* = 0.0102; Tukey's post hoc: C57BL/6J versus NEX (+/Cre) **p = 0.0074). Sample sizes of analyzed dendrites: C57BL/6J: *n* = 32, NEX (+/+): *n* = 13, NEX (+/Cre): *n* = 13, NEX (Cre/Cre): *n* = 17. (G) Increased spine density on neurons of the LSI in NEX (+/+) mice compared to C57BL/6J mice (Ordinary one‐way ANOVA: *F* (3, 62) = 6.124, *p* = 0.001; Tukey's post hoc: C57BL/6J versus NEX (+/+) ***p = 0.0005). Sample sizes of analyzed dendrites: C57BL/6J: *n* = 21, NEX (+/+): *n* = 18, NEX‐Cre (+/Cre): *n* = 13, NEX (Cre/Cre): *n* = 14. (H) Decreased spine density on apical dendrites of pyramidal neurons in the hippocampal CA1 region in NEX (Cre/Cre) compared to NEX (+/Cre) mice (Ordinary one‐way ANOVA: *F* (3, 87) = 2.43, *p* = 0.0705; Tukey's post hoc: NEX (+/Cre) versus NEX (Cre/Cre) *p = 0.0412). Sample sizes of analyzed dendrites: C57BL/6J: *n* = 19, NEX‐Cre (+/+): *n* = 25, NEX‐Cre (+/Cre): *n* = 28, NEX (Cre/Cre): *n* = 19. (I) Decreased spine density on basal dendrites of pyramidal neurons in the hippocampal CA1 region in NEX (Cre/Cre) compared to NEX (+/Cre), NEX (+/+) and C57BL/6J mice (Ordinary one‐way ANOVA: *F* (3, 107) = 12.98, *p* < 0.0001; Tukey's post hoc: C57BL/6J versus NEX (Cre/Cre) ****p < 0.0001, NEX (+/+) versus NEX (Cre/Cre) ****p < 0.0001, NEX (+/Cre) versus NEX(Cre/Cre) **p = 0.0019). Sample sizes of analyzed dendrites: C57BL/6J: *n* = 31, NEX (+/+): *n* = 30, NEX (+/Cre): *n* = 20, NEX (Cre/Cre): *n* = 30. (J) No differences in the dendritic spine density on neurons of the BLA between the groups (Ordinary one‐way ANOVA: *F* (3, 75) = 2.427, *p* = 0.0712). Sample sizes of analyzed dendrites: C57BL/6J: *n* = 17, NEX (+/+): *n* = 21, NEX (+/Cre): *n* = 16, NEX (Cre/Cre): *n* = 25. The total numbers of analyzed animals, brain slices and dendrites per region and genotype are provided in Table S1. The data is presented as individual data points on violin plot. The solid black line indicates the median, while the gray lines represent the 25th and 75th percentiles.

Given the abnormal behavioral traits observed in NEX‐Cre mice, such as anhedonic‐like behavior, reduced anxiety, and increased locomotion, and considering the critical role of NEX in neuronal development and survival, we investigated dendritic spine density in key brain regions associated with emotion, reward processing, decision‐making, learning, memory, motor planning, and social behavior. These regions included the medial prefrontal cortex (mPFC), nucleus accumbens core region (NaC), caudate putamen (CPu), dorsal (LSD) and intermediate (LSI) lateral septum, hippocampal CA1 region (Hip CA1), and basolateral amygdala (BLA). To assess dendritic spine density, Golgi‐Cox staining was performed and analyzed (Figure 4).

In the mPFC, NEX(+/+) mice showed increased spine density on apical dendrites compared to C57BL/6J and NEX(+/Cre) mice, with no differences observed on basal dendrites (Figure 4B,C). This unexpected increase suggests that external factors or subtle genetic variations may affect neural architecture in NEX (+/+) mice. In the NaC, spine density was elevated in NEX(Cre/Cre) mice compared to C57BL/6J and NEX (+/+) mice, and in NEX (+/Cre) mice compared to C57BL/6J mice (Figure 4D), indicating possible gene dosage effects of Cre recombinase or NEX deletion. In the CPu, all NEX‐Cre groups exhibited reduced spine density relative to C57BL/6J mice (Figure 4E). In the LSD and LSI, spine density was increased in NEX (+/Cre) and NEX (+/+) mice, respectively, compared to C57BL/6J mice (Figure 4F,G). In the hippocampal CA1 region, NEX (Cre/Cre) mice displayed reduced spine density on both apical and basal dendrites, with the latter reduction more pronounced across groups (Figure 4H,I). In the BLA, no significant group differences were observed, although NEX (Cre/Cre) mice showed a trend toward lower spine density (Figure 4J).

In summary, dendritic spine density was altered in a region‐ and genotype‐dependent manner in NEX‐Cre mice, indicating changes in synaptic architecture across multiple circuits. While increases were observed in select areas (e.g., mPFC and parts of the septum), reductions were particularly consistent in the CPu and most pronounced in the hippocampal CA1 region of NEX (Cre/Cre) mice. In contrast, no significant differences were detected in the BLA. Together, these findings suggest strain‐ and genotype‐specific shifts in synaptic connectivity that may contribute to the behavioral phenotypes observed in NEX‐Cre animals.

## 6 Discussion

### 6.1 Hyperlocomotion, Reduced Anxiety‐Like Behavior, Motor Dysfunctions, and Anhedonic‐Like Behavior in NEX(Cre/Cre) Mice

NEX (Cre/Cre) mice exhibited behavioral abnormalities, including increased exploration, hyperactivity, circling behavior, and reduced self‐grooming. These findings align with previous reports linking NEX knockout models to hyperlocomotion (Berg 2019; Mikhailova 2007). In contrast, NEX (+/Cre) mice showed no such abnormalities, suggesting compensation for NEX haploinsufficiency. Circling behavior, a stereotypy associated with basal ganglia dysfunction and dopaminergic dysregulation, and reduced self‐grooming, a behavior modulated by forebrain circuits, indicate disrupted neural systems in NEX (Cre/Cre) mice. Given that grooming is also an important measure in models of OCD and autism spectrum disorders (Kalueff et al. 2016), its absence may reflect broader neuropsychiatric‐like alterations, though motivational or competitive factors cannot be ruled out (Fitzgerald et al. 1991).

Reduced anxiety‐like behavior in NEX (Cre/Cre) mice is consistent with previous findings using the same model (Berg 2019), but contrasts with studies on NEX knockout mice lacking Cre expression (Mikhailova 2007), suggesting that Cre recombinase expression, in addition to NEX deletion, may influence the phenotype. However, it is important to note that the reduced anxiety levels observed in NEX (Cre/Cre) mutants may arise as a secondary effect of their hyperactivity like behavioral traits during anxiety related tests. Specifically, their increased presence in open areas, commonly interpreted as reduced anxiety, could instead reflect heightened locomotor activity rather than a genuine reduction in anxiety. Conversely, the increased locomotion observed in NEX(Cre/Cre) mutants could also be a direct result of reduced anxiety.

No major abnormalities in social interaction were observed across groups, though caution is warranted due to methodological limitations of the SI test, which may miss subtle social deficits. Future studies could employ more refined approaches, such as three‐chamber tests or machine learning‐based tracking (e.g., SLEAP (Pereira et al. 2022)), to capture detailed social behavior.

In the sucrose preference test, NEX (Cre/Cre) mice did not differ from C57BL/6J controls, indicating preserved sucrose preference relative to the true wild‐type background. In contrast, NEX (+/+) and NEX (+/Cre) mice showed reduced sucrose preference compared to C57BL/6J, and NEX (Cre/Cre) mice differed from their littermates. However, the absence of a clear shift relative to C57BL/6J argues against a robust anhedonia‐like phenotype. Instead, the increased overall fluid intake in NEX (Cre/Cre) mice points to altered consummatory drive, consistent with recent studies linking NEX‐positive VTA mDA neurons to consummatory aspects of reward‐related behavior (Bimpisidis et al. 2019, 2023).

### 6.2 Behavioral Classification by SVM

Despite mild and heterogeneous behavioral abnormalities in NEX‐Cre mice, SVM‐based classification successfully predicted genotypes using EPM and OFT data. While PCA and t‐SNE did not reveal a clear behavioral cluster, SVM achieved high accuracy, particularly for NEX (Cre/Cre) mice, consistent with their pronounced phenotype. Notably, the SVM also differentiated NEX (+/+) and (+/Cre) mice from C57BL/6J controls, revealing subtle behavioral traits in these genotypes. This challenges the assumption that NEX (+/Cre) mice fully compensate for haploinsufficiency.

Furthermore, distinguishing NEX (+/+) from C57BL/6J mice—despite identical genetic backgrounds—suggests possible maternal effects from NEX (+/Cre) dams, warranting future cross‐fostering studies. In contrast, genotype classification based on social interaction data failed (Figure S4), likely due to the limited behavioral parameters assessed.

Overall, our findings highlight motor and cognitive components in the hyperactivity‐like behavior of NEX (Cre/Cre) mice and reveal genotype‐specific traits in NEX (+/+) and (+/Cre) mice, emphasizing the influence of genetic background on behavior.

Consistent with established best practice in mouse genetics, our findings highlight that comparisons between a transgenic line and a separately bred inbred strain (such as C57BL/6J) are primarily descriptive and should not be used as a substitute for analyses based on wild‐type littermates. In particular, the observation that NEX (+/+) littermates do not phenotypically match C57BL/6J mice underscores the importance of using littermate controls rather than independently maintained or purchased C57BL/6J animals when interpreting genotype‐related effects.

### 6.3 Spine Density Alterations in NEX‐Cre Mice

Given the behavioral abnormalities observed in NEX‐Cre mice, we assessed dendritic spine density as a structural correlate of altered circuit function. Across regions, spine changes were strongly genotype‐ and circuit‐dependent, suggesting that Nex manipulation affects synaptic architecture in a nonuniform manner. In the NaC, increased spine density across NEX‐Cre genotypes suggests altered reward‐related circuitry, although behavioral outcomes did not follow a uniform pattern. Importantly, C57BL/6J mice represent the true wild‐type background control, and sucrose preference in NEX (+/+) animals was not significantly different from this baseline, indicating largely preserved consummatory behavior. In contrast, NEX (Cre/Cre) mice exhibited higher sucrose preference relative to NEX (+/+) and NEX (+/Cre) littermates despite showing the most pronounced increase in spine density. Together, these findings indicate that NaC spine density changes may contribute to altered reward processing, but their behavioral consequences appear genotype‐dependent and cannot be explained by a single mechanism across groups. Increased NaC spine density has been linked to anhedonia‐like behavior in chronic stress models (Bessa et al. 2013); however, our results suggest that similar structural changes may be associated with distinct behavioral outcomes depending on genotype and context.

In contrast, spine density was consistently reduced in the CPu across all NEX‐Cre groups, indicating a shared structural phenotype in striatal motor circuits (Grahn et al. 2008; Langen et al. 2011; Obeso and Lanciego 2011). However, overt motor abnormalities were largely restricted to NEX (Cre/Cre) mice, implying compensatory mechanisms in NEX (+/+) and NEX (+/Cre) animals that buffer functional consequences. In addition, motor behavior is shaped by distributed cortico–basal ganglia–thalamic and dopaminergic circuits, and alterations in other motor‐related regions may therefore contribute to the genotype‐specific behavioral differences observed here. The most pronounced reductions were observed in hippocampal CA1 dendrites of NEX (Cre/Cre) mice, consistent with the strong endogenous NEX expression in hippocampal circuits (Schwab et al. 1998) and potentially linked to previously reported cognitive impairments in NEX loss‐of‐function models (Mikhailova 2007). More selective, subregion‐specific increases in the lateral septum further support the idea of context‐dependent synaptic remodeling, possibly reflecting compensatory stabilization under partial NEXperturbation. Finally, the absence of significant spine density changes in the BLA suggests that anxiety‐related behavioral alterations in NEX (Cre/Cre) mice may arise from circuit mechanisms not captured by spine density alone.

Together, these findings highlight complex and region‐specific synaptic adaptations in NEX‐Cre mice that may contribute to the observed behavioral phenotypes, while also emphasizing that spine density changes can be compensatory or secondary rather than strictly causal. Future work combining spine morphology, circuit‐level connectivity, and functional readouts will be essential to clarify how NEX manipulation reshapes neural computations underlying reward, motor control, and cognition.

### 6.4 Genetical Background and Behavior

NEX is primarily expressed in glutamatergic principal neurons, and its haploinsufficiency or knockout may impair glutamatergic pathways, contributing to hyperactivity‐like behaviors. NEX is also expressed early during development (E14.5) in a subset of VTA dopaminergic (mDA) neurons projecting to the lateral septum (LS) and nucleus accumbens shell, regions involved in motor control and reward processing (Khan et al. 2017; Kramer et al. 2021, 2018; Bimpisidis et al. 2019, 2023; Dumas and Wallén‐Mackenzie 2019). NEX knockout studies demonstrate a reduction in VTA mDA neuron numbers, underscoring its essential role in dopaminergic development and survival (Khan et al. 2017).

The NEX‐Cre mouse model has been widely used to investigate neuronal development, signaling, and behavior (Bimpisidis et al. 2019, 2023; Mulder et al. 2008; Dedic et al. 2018; Keil et al. 2020; Karapinar et al. 2021; Miyoshi et al. 2021; Steubler et al. 2021; Loganathan et al. 2024) and has facilitated the creation of advanced genetic tools, such as the DAT‐P2A‐Flpo line for targeting NEX‐positive dopaminergic subpopulations (Kramer et al. 2021). However, the utility of NEX‐Cre mice depends on thorough characterization of their baseline phenotypes, as unrecognized behavioral or structural alterations could confound interpretations.

Importantly, knock‐in of Cre recombinase itself can induce ectopic gene expression, DNA damage, and behavioral abnormalities such as hyperactivity and impulsivity, independent of the targeted gene deletion (Schmidt et al. 2000; Desor et al. 2024; Lammel et al. 2015; Lindeberg et al. 2004; Kurachi et al. 2019). These side effects are often dose‐dependent and highlight the necessity of including appropriate Cre‐only controls with matched allele numbers in both behavioral and cellular studies. Relying solely on behavioral normality to exclude cellular or structural abnormalities is problematic, as subtle phenotypes may be overlooked.

Although inducible Cre systems (e.g., tamoxifen‐inducible) or viral strategies (e.g., CamKII promoters) offer alternatives to minimize developmental disturbances, they also have limitations. Thus, rigorous control strategies and advanced analytical approaches are crucial to accurately separate Cre‐related effects from true gene‐specific phenotypes, improving the reliability and interpretability of studies using Cre/loxP models.

An important finding of the present study is the lack of meaningful behavioral or structural differences between wild‐type NEX(+/+) and heterozygous NEX (Cre/+) mice. This observation indicates that a single functional NEX allele is sufficient to maintain normal behavioral performance and spine density and suggests that Cre recombinase expression from the NEX locus does not induce detectable nonspecific effects. Together, these results provide empirical support for the widely used practice of employing heterozygous NEX (Cre/+) mice in circuit‐specific genetic studies and reinforce the interpretation that neither loss of one NEX allele nor Cre expression from the NEX locus confounds phenotypic analyses under the conditions examined. An important limitation of the present work is the relatively broad age range of the animals (2–9 months), which may have contributed to inter‐individual variability in both behavioral measures and spine density. Future studies are also needed to determine whether the observed structural and behavioral alterations in NEX‐Cre mice extend to females, providing a more complete understanding of this model.

Overall, these considerations emphasize that careful experimental design, including comprehensive phenotypic validation, is essential when using NEX‐Cre mice or related models to ensure accurate conclusions about gene function and neuronal circuitry.

## Author Contributions

**Kim Renken:** conceptualization, writing – original draft, methodology, validation, writing – review and editing, formal analysis, visualization, investigation. **Olivia Andrea Masseck:** conceptualization, writing – original draft, writing – review and editing, software, supervision, resources, funding acquisition.

## Disclosure

During the preparation of this work, the author(s) used ChatGPT in order to improve language and to assist in designing the data analysis for the SVM. After using this tool or service, the author(s) reviewed and edited the content as needed and takes full responsibility for the content of the publication.

## Conflicts of Interest

Olivia Masseck is a Handling Editor of JNC.

## Supporting information


**Appendix S1:** jnc70401‐sup‐0001‐AppendixS1.pdf.

## Data Availability

This study did not generate new unique reagents. A version of this manuscript was posted on bioRxiv; 21/05/2025; https://doi.org/10.1101/2025.05.21.655260. Data reported in this paper and the utilized code for NEX‐Cre classification‐ can be found at [https://github.com/masseck]. Any additional information required to reanalyze the data reported in this paper is available from the lead contact upon request.
