# Supplementary material for: Uncovering Hidden Phenotypes in NEX‐Cre Mice: Behavioral and Cellular Alterations Demand Re‐Evaluation of a Widely Used Transgenic Line
Source: J Neurochem. 2026 Mar 15;170(3):e70401. doi: 10.1111/jnc.70401 (PMC12989317; doi:10.1111/jnc.70401)
Supplement: Supplementary file 1 — Appendix S1: jnc70401‐sup‐0001‐AppendixS1.pdf. [file JNC-170-0-s001.pdf]

Title: Uncovering Hidden Phenotypes in NEX-Cre Mice: Behavioral and Cellular Alterations Demand Re-Evaluation of a Widely Used Transgenic Line

Kim Renken[1] & Olivia Andrea Masseck[1,2]

[1] Synthetic Biology, University of Bremen, Bremen, Germany

[2] Neuromodulatory Circuits, University of Cologne, Cologne, Germany

Supplementary Information

Table S1: Summary of the total number of animals, brain slices, and dendrites analyzed for each brain region and genotype included in the spine density analysis

brain region	group	mice (n)	brain slices (n)	dendrites (n)
**CPU**	C57BL/6J	3	6	17
	NEX-Cre (wt/wt)	3	4	21
	NEX-Cre (wt/+)	4	6	16
	NEX-Cre (+/+)	3	7	25
**NaC**	C57BL/6J	3	5	17
	NEX-Cre (wt/wt)	3	6	15
	NEX-Cre (wt/+)	4	10	38
	NEX-Cre (+/+)	3	3	18
**CA1 apical**	C57BL/6J	3	6	19
	NEX-Cre (wt/wt)	4	4	25
	NEX-Cre (wt/+)	5	7	28
	NEX-Cre (+/+)	3	4	19
**CA1 basal**	C57BL/6J	3	7	31
	NEX-Cre (wt/wt)	4	4	30
	NEX-Cre (wt/+)	4	5	20
	NEX-Cre (+/+)	3	4	31
**LSD**	C57BL/6J	3	3	32
	NEX-Cre (wt/wt)	4	4	13
	NEX-Cre (wt/+)	4	2	13
	NEX-Cre (+/+)	4	4	17
**LSI**	C57BL/6J	3	3	21
	NEX-Cre (wt/wt)	4	3	18
	NEX-Cre (wt/+)	4	2	13
	NEX-Cre (+/+)	3	4	14
**mPFC apical**	C57BL/6J	3	7	33
	NEX-Cre (wt/wt)	5	10	25
	NEX-Cre (wt/+)	4	10	32
	NEX-Cre (+/+)	4	7	15
**mPFC basal**	C57BL/6J	3	6	52
	NEX-Cre (wt/wt)	5	8	26
	NEX-Cre (wt/+)	6	11	44
	NEX-Cre (+/+)	3	6	21

Table S2: Sample size for all Behavioral Experiments

	C57BL/6J	NEX(+/+)	NEX(Cre/+)	NEX(Cre/Cre)	total number #
**EPM**	20	17	22	26	85
**OFT**	20	16	22	28	86
**NSFT**	11	10	11	22	54
**SPT**	16	16	22	12	66

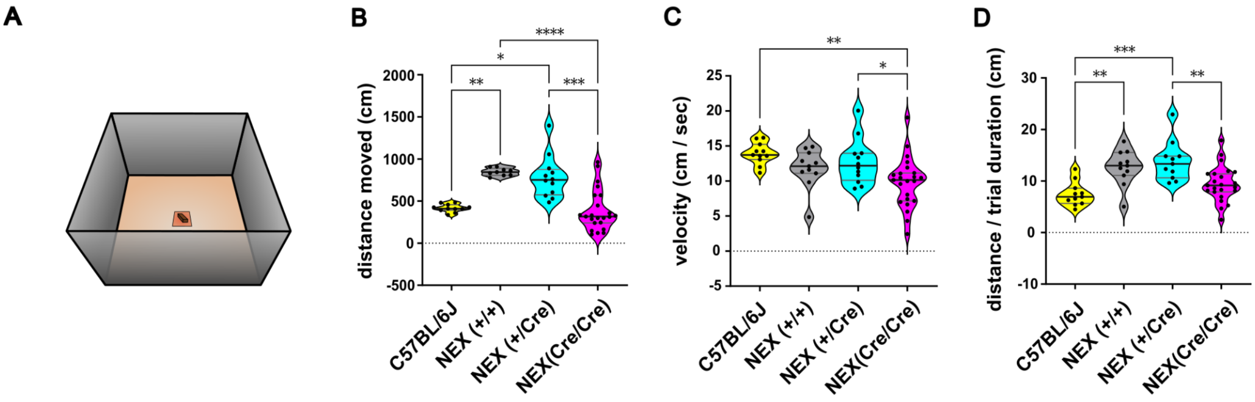

Figure S1: Distance moved and Velocity in NSFT. **(**A) Schematic overview of the NSFT. (B) NEX (Cre/Cre) covered significantly less distance compared to NEX (+/+) and NEX (+/Cre) mice. NEX (+/+) and NEX (+/Cre) covered significantly more distance compared to C57BL/6J (Kruskal Wallis ANOVA: H (3) = 28.98, p < 0.0001; Dunn`s post hoc: NEX (Cre/Cre) vs NEX +/+) **** p < 0.0001, NEX (Cre/Cre) vs NEX(+/Cre) ***p = 0.0005, NEX(+/+) vs C57BL/6J **p = 0.0067, NEX (+/Cre) vs C57BL/6J *p = 0.0446). Sample sizes: C57BL/6J: n = 11, NEX (+/+): n = 9, NEX (+/Cre): n = 12, NEX (Cre/Cre): n = 23. (C) NEX (Cre/Cre) showed significantly lower mean velocity than C57BL/6J and NEX (+/Cre) (Ordinary one-way ANOVA: F (3,53) = 5.332, p = 0.0028; Tukey`s post hoc: C57BL/6J vs NEX (Cre/Cre) **p = 0.003, NEX (+/Cre) vs NEX (Cre/Cre) *p = 0.046).Sample sizes: C57BL/6J: n = 11, NEX (+/+): n = 11, NEX (+/Cre): n = 12, NEX (Cre/Cre): n = 23. (D) For normalization, the distance covered in the NSFT is divided by the respective latency to consume food. Relative to the trail duration, the NEX (Cre/Cre) have travelled less distance than the NEX (+/Cre). The NEX (+/+) and the NEX (+/Cre) have travelled more distance relative to the C57BL/6J (Ordinary one-way ANOVA: F (3,52) = 8.933, ****p < 0.0001; Tukey`s post hoc: C57BL/6J vs NEX (+/+) **p = 0.0034, C57BL/6J vs NEX (+/Cre) ***p = 0.0002, NEX (+/Cre) vs NEX (Cre/Cre) **p = 0.0043). Sample sizes: C57BL/6J: n = 11, NEX (+/+): n = 11, NEX (+/Cre): n = 11, NEX (Cre/Cre): n = 23.The data is presented as individual data points on violin plot. The solid black line indicates the median, while the grey lines represent the 25th and 75th percentiles.

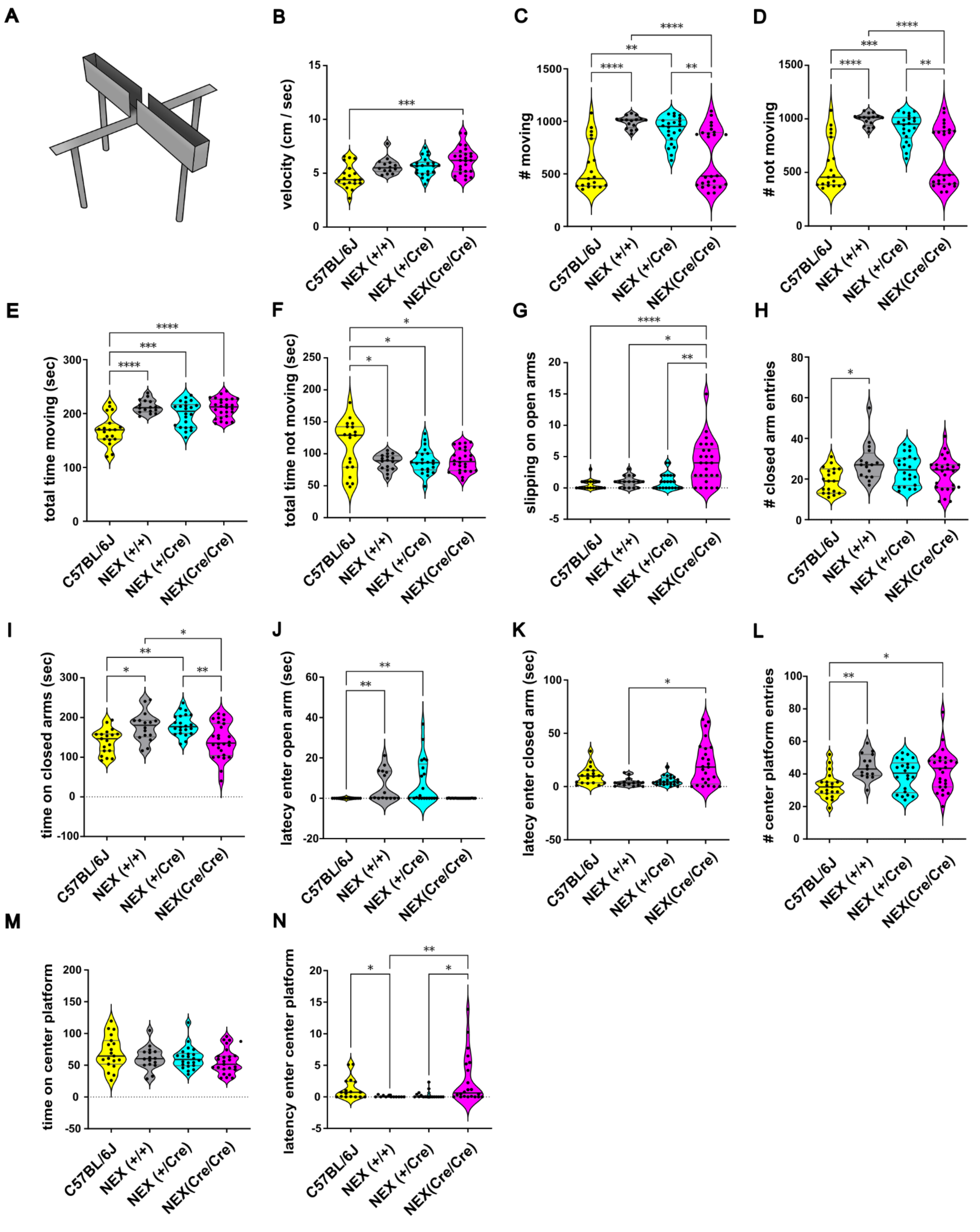

Figure S2: Additional parameters measured in the EPM that were also included in the SVM analysis.
(A) Schematic overview of the EPM. (B) NEX (Cre/Cre) showed significantly increased mean velocity than

C57BL/6J (Kruskal Wallis ANOVA: H (3) = 16.11, **p = 0.0011; Dunn`s post hoc: C57BL/6J vs NEX (Cre/Cre) *** p = 0.0004). Sample sizes: C57BL/6J: n = 19, NEX (+/+): n = 14, NEX (+/Cre): n = 22, NEX (Cre/Cre): n = 26. (C) NEX (Cre/Cre) showed a significantly lower movement frequency than NEX (+/+) and NEX (+/Cre). NEX (+/+) and NEX (+/Cre) showed a significantly higher movement frequency than C57BL/6J (Kruskal Wallis ANOVA: H (3) = 33.46, ****p < 0.0001; Dunn`s post hoc: NEX (Cre/Cre) vs NEX (+/+) **** p < 0.0001, NEX (Cre/Cre) vs NEX (+/Cre) ** p = 0.0083, C57BL/6J vs NEX (+/+) **** p < 0.0001, C57BL/6J vs NEX (+/Cre) ** p = 0.0011). Sample sizes: C57BL/6J: n = 19, NEX (+/+): n = 16, NEX (+/Cre): n = 22, NEX (Cre/Cre): n = 26. (D) Consequently, NEX (Cre/Cre) showed a significantly lower frequency not moving than NEX (+/+) and NEX (+/Cre). NEX (+/+) and NEX (+/Cre) showed a significantly higher frequency not moving than C57BL/6J (Kruskal Wallis ANOVA: H (3) = 33.47, ****p < 0.0001; Dunn`s post hoc: NEX (Cre/Cre) vs NEX (+/+) **** p < 0.0001, NEX (Cre/Cre) vs NEX (+/Cre) ** p = 0.0078, C57BL/6J vs NEX (+/+) **** p < 0.0001, C57BL/6J vs NEX (+/Cre) *** p = 0.0010). Sample sizes: C57BL/6J: n = 19, NEX (+/+): n = 16, NEX (+/Cre): n = 22, NEX (Cre/Cre): n = 26. (E) NEX (+/+), NEX (+/Cre) and NEX (Cre/Cre) spent significantly more time moving compared to C57BL/6J mice. (Ordinary one-way ANOVA: F (3,79) = 17.29, ****p < 0.0001; Tukey`s post hoc: C57BL/6J vs NEX (+/+) ****p < 0.0001, C57BL/6J vs NEX (+/Cre) *** p = 0.0002, C57BL/6J vs NEX (Cre/Cre) ****p < 0.0001). Sample sizes: C57BL/6J: n = 19, NEX (+/+): n = 16, NEX (+/Cre): n = 22, NEX (Cre/Cre): n = 26. (F) Consequently, NEX (+/+), NEX (+/Cre) and NEX (Cre/Cre) spent significantly less time not moving compared to C57BL/6J mice (Ordinary one-way ANOVA: F (3,79) = 4.420, **p = 0.0063; Tukey`s post hoc: C57BL/6J vs NEX (+/+) *p = 0.0196, C57BL/6J vs NEX (+/Cre) * p = 0.0168, C57BL/6J vs NEX (Cre/Cre) *p = 0.0186). Sample sizes: C57BL/6J: n = 19, NEX (+/+): n = 16, NEX (+/Cre): n = 22, NEX (Cre/Cre): n = 26. (G) NEX (Cre/Cre) were significantly more likely to slip their hind paws off the open arms compared to NEX (+/+), NEX (+/Cre) and C57BL/6J mice (Kruskal Wallis ANOVA: H (3) = 23.29, ****p < 0.0001; Dunn`s post hoc: C57BL/6J vs NEX (Cre/Cre) **** p < 0.0001, NEX (+/+) vs NEX (Cre/Cre) * p = 0.0237, NEX (+/Cre) vs NEX (Cre/Cre) ** p = 0.0034). Sample sizes: C57BL/6J: n = 19, NEX (+/+): n = 16, NEX (+/Cre): n = 22, NEX (Cre/Cre): n = 26. (H) NEX (+/+) entered the closed arms significantly more often compared to C57BL/6J mice (Kruskal Wallis ANOVA: H (3) = 10.67, *p = 0.0136; Dunn`s post hoc: C57BL/6J vs NEX (+/+) * p = 0.0105). Sample sizes: C57BL/6J: n = 19, NEX (+/+): n = 16, NEX (+/Cre): n = 22, NEX (Cre/Cre): n = 26. (I) NEX (Cre/Cre) spent significant less time on closed arms compared to NEX (+/Cre) and NEX (+/+) while NEX (+/Cre) and NEX (+/+) spent significantly more time on closed arms compared to C57BL/6J mice (Ordinary one-way ANOVA: F (3,79) = 7.976, ***p = 0.0001; Tukey`s post hoc: C57BL/6J vs NEX (+/+) *p = 0.0183, C57BL/6J vs NEX (+/Cre) **p = 0.0025, NEX (Cre/Cre) vs NEX (+/+) *p = 0.0140, NEX (Cre/Cre) vs NEX (+/Cre) **p = 0.0014). Sample sizes: C57BL/6J: n = 19, NEX (+/+): n = 16, NEX (+/Cre): n = 22, NEX (Cre/Cre): n = 26. (J) NEX (+/+) and NEX (+/Cre) had a significantly increased latency to enter the open arms compared to C57BL/6J mice. (Kruskal Wallis ANOVA: H (3) = 18.00, ***p = 0.0004; Dunn`s post hoc: C57BL/6J vs NEX (+/+) **p = 0.0058, C57BL/6J vs NEX (+/Cre) **p = 0.0049). Sample sizes: C57BL/6J: n = 14, NEX (+/+): n = 15, NEX (+/Cre): n = 21, NEX (Cre/Cre): n = 18. (K) NEX (Cre/Cre) showed a significantly increased latency to enter closed arms compared to NEX (+/+) (Kruskal Wallis ANOVA: H (3) = 12.39, **p = 0.0062; Dunn`s post hoc: NEX (+/+) vs NEX (Cre/Cre) *p = 0.0124). Sample sizes: C57BL/6J: n = 18, NEX (+/+): n = 15, NEX (+/Cre): n = 22, NEX (Cre/Cre): n = 25. (L) NEX (Cre/Cre) and NEX (+/+) showed significantly increased center platform entries compared to C57BL/6J mice. (Ordinary one-way ANOVA: F (3,78) = 4,737, **p = 0.0043; C57BL/6J vs NEX (+/+) **p = 0.0058, C57BL/6J vs NEX (Cre/Cre) *p = 0.0137). Sample sizes: C57BL/6J: n = 19, NEX (+/+): n = 15, NEX (+/Cre): n = 22, NEX (Cre/Cre): n = 26. (M) All groups spent a similar amount of time on the center platform (Kruskal Wallis ANOVA: H (3) = 4,367, ns p = 0.2245): Sample sizes: C57BL/6J: n = 19, NEX (+/+): n = 16, NEX (+/Cre): n = 22, NEX (Cre/Cre): n = 25. (N) NEX (Cre/Cre) had a significantly increased latency to enter the center platform compared to NEX (+/+) and NEX (+/Cre) mice, while C57BL/6J had a significantly increased latency to enter the center platform compared to NEX (+/+) mice (Kruskal Wallis ANOVA: H (3) = 14.99, **p = 0.0018; Dunn`s post hoc: C57BL/6J vs NEX (+/+) *p = 0.0498, NEX (+/+) vs NEX (Cre/Cre) **p = 0.0072, NEX (+/Cre) vs NEX (Cre/Cre) *p = 0.0396). Sample sizes: C57BL/6J: n = 17, NEX (+/+): n = 12, NEX (+/Cre): n = 17, NEX (Cre/Cre): n = 22.The data is presented as individual data points on violin plot. The solid black line indicates the median, while the grey lines represent the 25th and 75th percentiles.

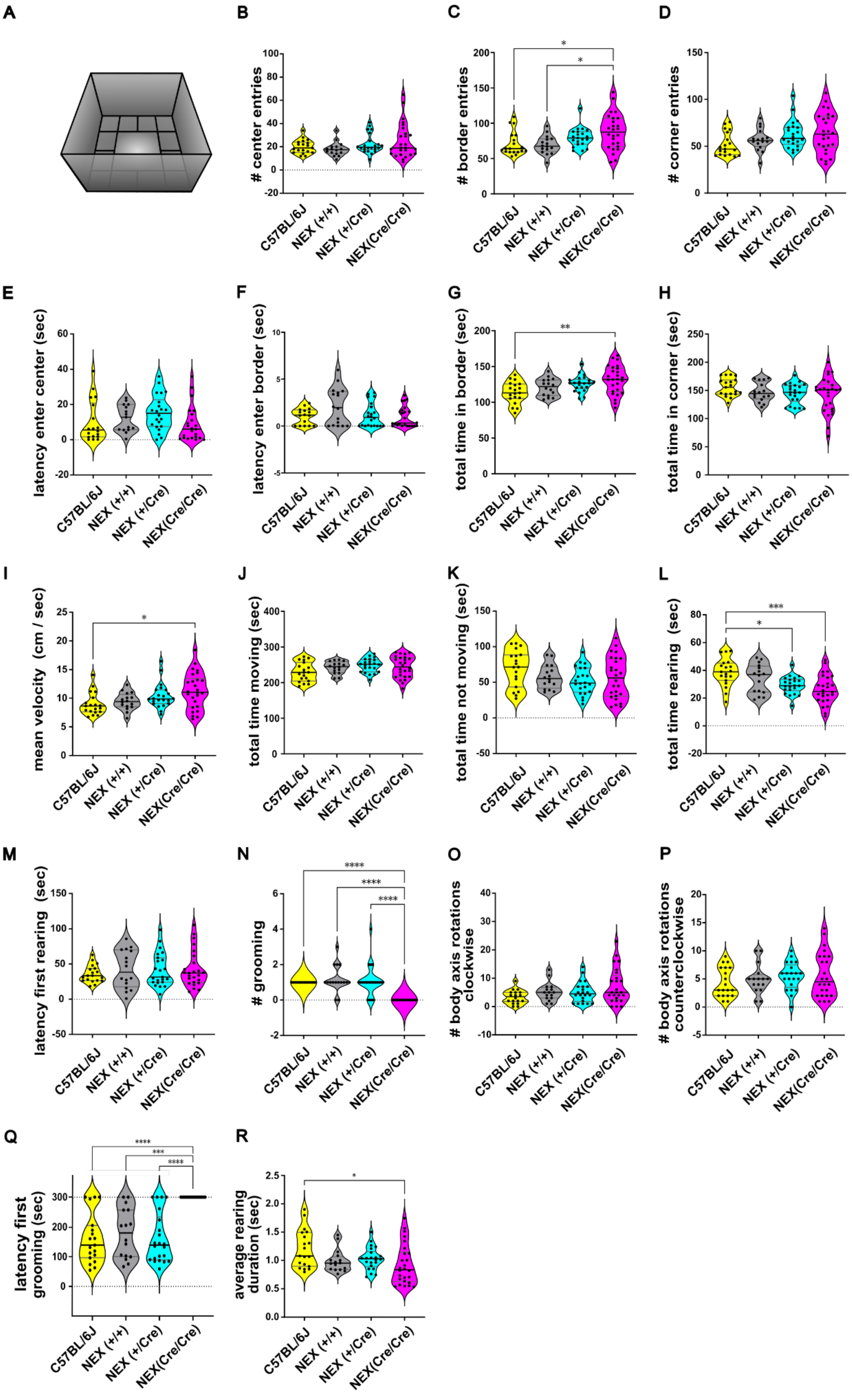

A
B
C
D
E
F
G
H
I
J
K
L
M
N
O
P
Q
R
center entries
border entries
corner entries
latency enter center (sec)
latency enter border (sec)
total time in border (sec)
total time in corner (sec)
mean velocity (cm / sec)
total time moving (sec)
total time not moving (sec)
total time rearing (sec)
latency first rearing (sec)
grooming
body axis rotations clockwise
body axis rotations counterclockwise
latency first grooming (sec)
average rearing duration (sec)
C57BL/6J
NEX (+/+)
NEX (+/Cre)
NEX(Cre/Cre)

Figure S3 : Additional parameters measured in the OFT that were also included in the SVM analysis. (A) Schematic overview of the EPM. (B) All groups entered the center field with similar frequency (Kruskal Wallis ANOVA: H (3) = 3.586, ns p = 0.3098). Sample sizes: C57BL/6J: n = 19, NEX (+/+): n = 16, NEX (+/Cre): n = 22, NEX (Cre/Cre): n = 26.(C) NEX (Cre/Cre) entered significantly more often the border zone compared to NEX (+/+) and C57BL/6J mice (Kruskal Wallis ANOVA: H (3) = 12,47, ** p = 0.0059; Dunn`s post hoc: NEX (Cre/Cre) vs C57BL/6J *p = 0.02, NEX (Cre/Cre) vs NEX (+/+) *p = 0.0435). Sample sizes: C57BL/6J: n = 19, NEX (+/+): n = 16, NEX (+/Cre): n = 21, NEX (Cre/Cre): n = 26. (D) All groups entered the corner zones with similar frequency (Kruskal Wallis ANOVA: H (3) = 7.511, ns p = 0.0573). Sample sizes: C57BL/6J: n = 19, NEX (+/+): n = 16, NEX (+/Cre): n = 22, NEX (Cre/Cre): n = 26. (E) All groups entered the center field after a similar latency (Kruskal Wallis ANOVA: H (3) = 7.393, ns p = 0.0604). Sample sizes: C57BL/6J: n = 18, NEX (+/+): n = 15, NEX (+/Cre): n = 22, NEX (Cre/Cre): n = 23. (F) All groups entered the border zone after a similar latency (Kruskal Wallis ANOVA: H (3) = 3.191, ns p = 0.3631). Sample sizes: C57BL/6J: n = 16, NEX (+/+): n = 16, NEX (+/Cre): n = 22, NEX (Cre/Cre): n = 24. (G) NEX (Cre/Cre) spent significantly more time in border zones than C57BL/6J mice (Ordinary one-way ANOVA: F (3,79) = 5.323, **p = 0.0022; Tukey`s post hoc: NEX (Cre/Cre) vs C57BL/6J **p = 0.0012). Sample sizes: C57BL/6J: n = 19, NEX (+/+): n = 16, NEX (+/Cre): n = 22, NEX (Cre/Cre): n = 26. (H) All groups spent similar time in corner zones (Ordinary one-way ANOVA: F (3,79) = 1.423, ns p = 0.2422). Sample sizes: C57BL/6J: n = 19, NEX (+/+): n = 16, NEX (+/Cre): n = 22, NEX (Cre/Cre): n = 26. (I) NEX (Cre/Cre) had a significantly increased mean velocity compared to C57BL/6J mice (Kruskal Wallis ANOVA: H (3) = 10.33, * p = 0.0160; Dunn`s post hoc: NEX (Cre/Cre) vs C57BL/6J *p = 0.0157). Sample sizes: C57BL/6J: n = 19, NEX (+/+): n = 16, NEX (+/Cre): n = 22, NEX (Cre/Cre): n = 26. (J) All groups spent similar time moving (Ordinary one-way ANOVA: F (3,79) = 2.12, ns p = 0.1043). Sample sizes: C57BL/6J: n = 19, NEX (+/+): n = 16, NEX (+/Cre): n = 22, NEX (Cre/Cre): n = 26. (K) All groups spent similar time not moving (Ordinary one-way ANOVA: F (3,79) = 1.959, ns p = 0.127). Sample sizes: C57BL/6J: n = 19, NEX (+/+): n = 16, NEX (+/Cre): n = 22, NEX (Cre/Cre): n = 26. (L) NEX (Cre/Cre) and NEX (+/Cre) mice spent significantly more time rearing compared to C57BL/6J (Ordinary one-way ANOVA: F (3,79) = 7.212, ***p = 0.0002; Tukey`s post hoc: NEX (Cre/Cre) vs C57BL/6J ***p = 0.0002, NEX (+/Cre) vs C57BL/6J *p = 0.0101). Sample sizes: C57BL/6J: n = 19, NEX (+/+): n = 16, NEX (+/Cre): n = 22, NEX (Cre/Cre): n = 26. (M) All groups had a similar latency to start rearing (Kruskal Wallis ANOVA: H (3) = 0.2406, ns p = 0.9708). Sample sizes: C57BL/6J: n = 19, NEX (+/+): n = 16, NEX (+/Cre): n = 23, NEX (Cre/Cre): n = 25. (N) NEX (Cre/Cre) exhibited a significantly reduced frequency of self-grooming events compared to C57BL/6J, NEX (+/+) and NEX (+/Cre) mice (Kruskal Wallis ANOVA: H (3) = 41.82, ****p < 0.0001; Dunn`s post hoc: NEX (Cre/Cre) vs C57BL/6J ****p < 0.0001, NEX (Cre/Cre) vs NEX (+/+) ****p < 0.0001, NEX (Cre/Cre) vs NEX (+/Cre) ****p < 0.0001). Sample sizes: C57BL/6J: n = 14, NEX (+/+): n = 16, NEX (+/Cre): n = 22, NEX (Cre/Cre): n = 20. (O) All groups showed a similar frequency of body axis rotations in a clockwise direction (Kruskal Wallis ANOVA: H (3) =5.051, ns p = 0.1681). Sample sizes: C57BL/6J: n = 18, NEX (+/+): n = 16, NEX (+/Cre): n = 22, NEX (Cre/Cre): n = 25. (P) All groups showed a similar frequency of body axis rotations in a counterclockwise direction (Kruskal Wallis ANOVA: H (3) = 2.165, ns p = 0.5388). Sample sizes: C57BL/6J: n = 19, NEX (+/+): n = 16, NEX (+/Cre): n = 21, NEX (Cre/Cre): n = 24. (Q) Illustration of the latency in seconds until animals initiated self-grooming behavior during OFT. The total test duration was 300 seconds, and animals that did not exhibit grooming behavior are represented by data points at the 300-second mark. As no self-grooming was observed in NEX (Cre/Cre), the latency is significantly longer than in the other groups. There is no significant difference in the latency of first grooming between the other groups (Kruskal Wallis ANOVA: H (3) = 33.03, ****p < 0.0001; Dunn`s post hoc: NEX (Cre/Cre) vs C57BL/6J ****p < 0.0001, NEX (Cre/Cre) vs NEX (+/+) ***p = 0.0002, NEX (Cre/Cre) vs NEX (+/Cre) ****p < 0.0001). Sample sizes: C57BL/6J: n = 19, NEX (+/+): n = 16, NEX (+/Cre): n = 22, NEX (Cre/Cre): n = 20. (R) Compared to C57BL/6J mice, the NEX (Cre/Cre) spent significantly less time per rearing (Kruskal Wallis ANOVA: H (3) = 9.214, ****p = 0.0266; Dunn`s post hoc: NEX (Cre/Cre) vs C57BL/6J *p = 0.0201). Sample sizes: C57BL/6J: n = 19, NEX (+/+): n = 16, NEX (+/Cre): n = 22, NEX (Cre/Cre): n = 20.The data is presented as individual data points on violin plot. The solid black line indicates the median, while the grey lines represent the 25th and 75th percentiles.

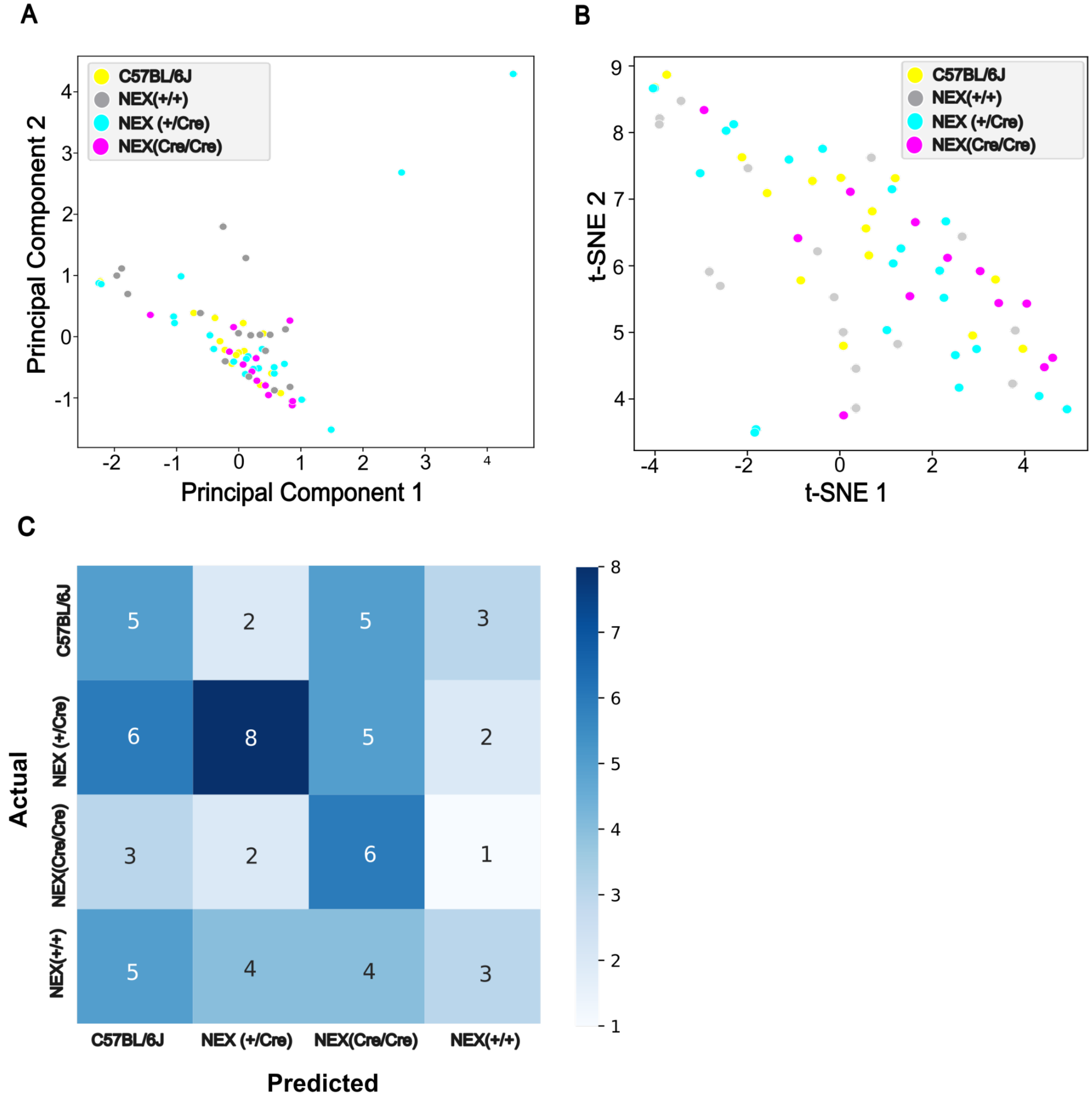

Figure S 4: Dimensionality Reduction and Classification Analysis of Behavioral Data from Social Interaction Test: PCA, t-SNE, SVM Confusion Matrix. (A) Visualization of the dataset after transformation and dimensionality reduction using PCA, highlighting the primary patterns and capturing 100% of the variance in the dataset. B) Visualization of the dataset preprocessed by PCA and then transformed and reduced to two dimensions using t-SNE, with a silhouette score of -0.1869 indicating strong cluster overlap, and a trustworthiness score of 0.9125 reflecting a high level of preservation of local data structure. (C) Visualization of the SVM's prediction performance, with an average accuracy of 34.23% across 5 cross-validation folds, as shown in an aggregated confusion matrix. The model's accuracy is not significantly above the chance rate of 32.81%, as indicated by a binomial test (^{ns} p = 0.5469). Sample sizes: C57BL/6J: n = 15, NEX (+/+): n = 16, NEX (+/Cre): n = 22, NEX (Cre/Cre): n =12.
